# Supramolecular coordination platinum metallacycle–based multilevel wound dressing for bacteria sensing and wound healing

**DOI:** 10.1073/pnas.2318391121

**Published:** 2024-03-25

**Authors:** Wen-Zhen Li, Xiao-Qiang Wang, Ling-Ran Liu, Ju Xiao, Xin-Qiong Wang, Yu-Yuan Ye, Zi-Xin Wang, Mai-Yong Zhu, Yao Sun, Peter J. Stang, Yan Sun

**Affiliations:** ^a^Interdisciplinary Institute of NMR and Molecular Sciences, Key Laboratory of Coal Conversion and New Carbon Materials of Hubei Province, School of Chemistry and Chemical Engineering, Wuhan University of Science and Technology, Wuhan 430081, China; ^b^Key Laboratory for Special Functional Materials of Ministry of Education, School of Materials Science and Engineering, Collaborative Innovation Center of Nano Functional Materials and Applications, Henan University, Zhengzhou 450046, China; ^c^Research School of Polymeric Materials, School of Materials Science and Engineering, Jiangsu University, Zhenjiang 212013, China; ^d^Department of Paediatrics, Ruijin Hospital, School of Medicine, Shanghai Jiao Tong University, Shanghai 200240, China; ^e^Key Laboratory of Pesticides and Chemical Biology, Ministry of Education, College of Chemistry, Central China Normal University, Wuhan 430079, China; ^f^Department of Chemistry, University of Utah, Salt Lake City, UT 84112

**Keywords:** coordination assembly, macroscopic supramolecular materials, wound healing

## Abstract

The exploitation of novel wound healing methods with real-time infection sensing and high spatiotemporal precision is highly important for human health. Pt-based metal-organic cycles/cages (MOCs) have been employed as multifunctional antibacterial agents due to their superior Pt-related therapeutic efficiency, various functional subunits and specific geometries. However, how to rationally apply these nanoscale MOCs on the macroscale with controllable therapeutic output is still challenging. Here, a centimeter-scale Pt MOC film was constructed via multistage assembly and subsequently coated on a N,N′-dimethylated dipyridinium thiazolo[5,4-d]thiazole (MPT)-stained silk fabric to form a smart wound dressing for bacterial sensing and wound healing. The MPT on silk fabric could be used to monitor wound infection in real-time through the bacteria-mediated reduction of MPT to its radical form via a color change. The MPT radical also exhibited an excellent photothermal effect under 660 nm light irradiation, which could not only be applied for photothermal therapy but also induce the disassembly of the Pt MOC film suprastructure. The highly ordered Pt MOC film suprastructure exhibited high biosafety, while it also showed improved antibacterial efficiency after thermally induced disassembly. In vitro and in vivo studies revealed that the combination of the Pt MOC film and MPT-stained silk can provide real-time information on wound infection for timely treatment through noninvasive techniques. This study paves the way for bacterial sensing and wound healing with centimeter-scale metal-organic materials.

## Significance

Non-healing wounds induced by antibiotic resistance have emerged as serious threats to human health. As promising candidates for the treatment of bacterial infections, metal-organic cycles/cages (MOCs) exhibit excellent antibacterial properties. However, the rational application of nanoscale MOCs has been limited due to difficulties in processability and transferability. To address these problems, a centimeter-scale Pt MOC film was constructed via multistage assembly and improved by coating it on N,N′-dimethylated dipyridinium thiazolo[5,4-d]thiazole (MPT)-stained silk fabric for bacterial sensing and wound healing. The as-prepared multilevel wound dressing enabled the monitoring of wound infection in real time and timely treatment with high spatiotemporal precision.

Bacterial infection associated with antibiotic resistance is an increasing threat to global health (1–6); in particular, without timely diagnosis and treatment, wounds may be invaded by pathogens, leading to chronic wound and tissue damage (7, 8). Therefore, the development of methods for timely monitoring of bacterial infection and treatment through noninvasive techniques is urgently needed (9–12). Currently, the traditional routes of drug administration are oral and parenteral, but these methods have clear disadvantages, such as gastrointestinal irritation and first-pass inactivation, which cannot resolve side effects (13). As an alternative method, topical treatment is a fast and direct method of drug delivery to the target tissue region that can prevent systemic reactions, reduce side effects, and achieve real-time infection monitoring (14). Recently, supramolecular coordination compounds (SCCs) (15–25), such as Pt MOCs (metal-organic cycles/cages) (26–30), have exhibited excellent properties as multifunctional antibacterial agents (31–36). Nevertheless, little is known about how to rationally exploit SCC materials for topical treatment. In our previous work, the first Pt MOC-based macroscopic films with multilevel hierarchical suprastructures were generated (37), which integrated the emissive properties of individual Pt MOCs and amplified the photophysical signal (38, 39).

Here, oligo (ethylene glycol) (OEG)-modified soft SCC films on a centimeter scale were designed and synthesized via multistage assembly. The centimeter-scale Pt MOC film with multilevel suprastructures obtained by multistage assembly had a loading capacity of 300%, which is far greater than that obtained via direct Pt MOC treatment. This excellent performance stems from the high specific surface area of the multilevel structures formed by the Pt MOCs. Moreover, upon disassembly of the centimeter-scale films with stimulus-responsive properties, Pt MOCs are released and can fully contact the bacteria and directly act on the bacterial surface, producing antibacterial effects and reducing the toxicity and side effects of Pt MOCs, thus making these centimeter-scale films potential smart wound dressing candidates. In particular, N,N′-dimethylated dipyridinium thiazolo[5,4-d]thiazole (MPT)-stained silk fabric (40–46) exhibited a change in color from slightly yellow to deep purple after cocultivation with bacteria, indicating its potential as a smart wound dressing for monitoring bacterial infection in real-time.

**Scheme 1.**
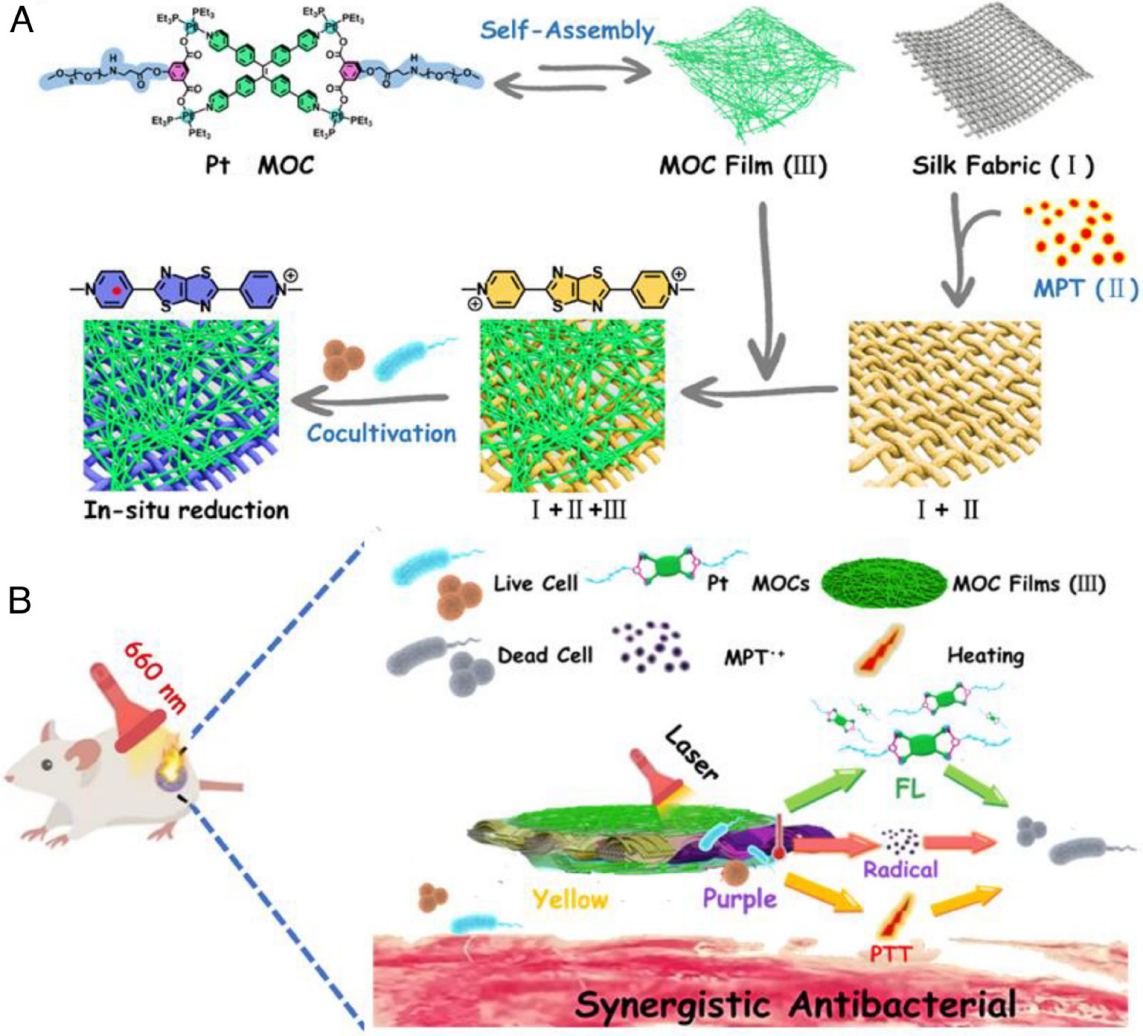
(*A*) Structures of Pt MOCs and MPT, the self-assembly and disassembly of a centimeter Pt MOC film (III), and the bacteria-mediated reduction of MPT(II) to its radical form. (*B*) Schematic diagram of the antibacterial mechanism of complex I+II+III in vivo.

As shown in Scheme 1, silk fabric (I) stained with MPT (II) and coated with a centimeter of Pt MOC film (III) was prepared as a multifunctional wound dressing (I+II+III) for real-time infection monitoring and wound healing. Self-assembled Pt MOCs can form centimeter-scale films with good portability and biosafety (Scheme 1*A*). The radical form of MPT can be reduced by bacteria, which results in a color change and can enable real-time monitoring of infection. Silk fabric (I), as a substrate, was employed to enhance the strength of the Pt MOC film and stabilize the MPT radical (Scheme 1*B*). The as-prepared wound dressing (I+II+III) exhibited a color change after being cocultured with bacteria along with a remarkable photothermal effect under 660 nm light irradiation, which promoted the disassembly of the Pt MOC film suprastructure with improved antibacterial efficiency. This smart wound dressing was successfully applied for bacterial sensing and wound healing in animal models.

## Results and Discussion

**Fig. 1.**
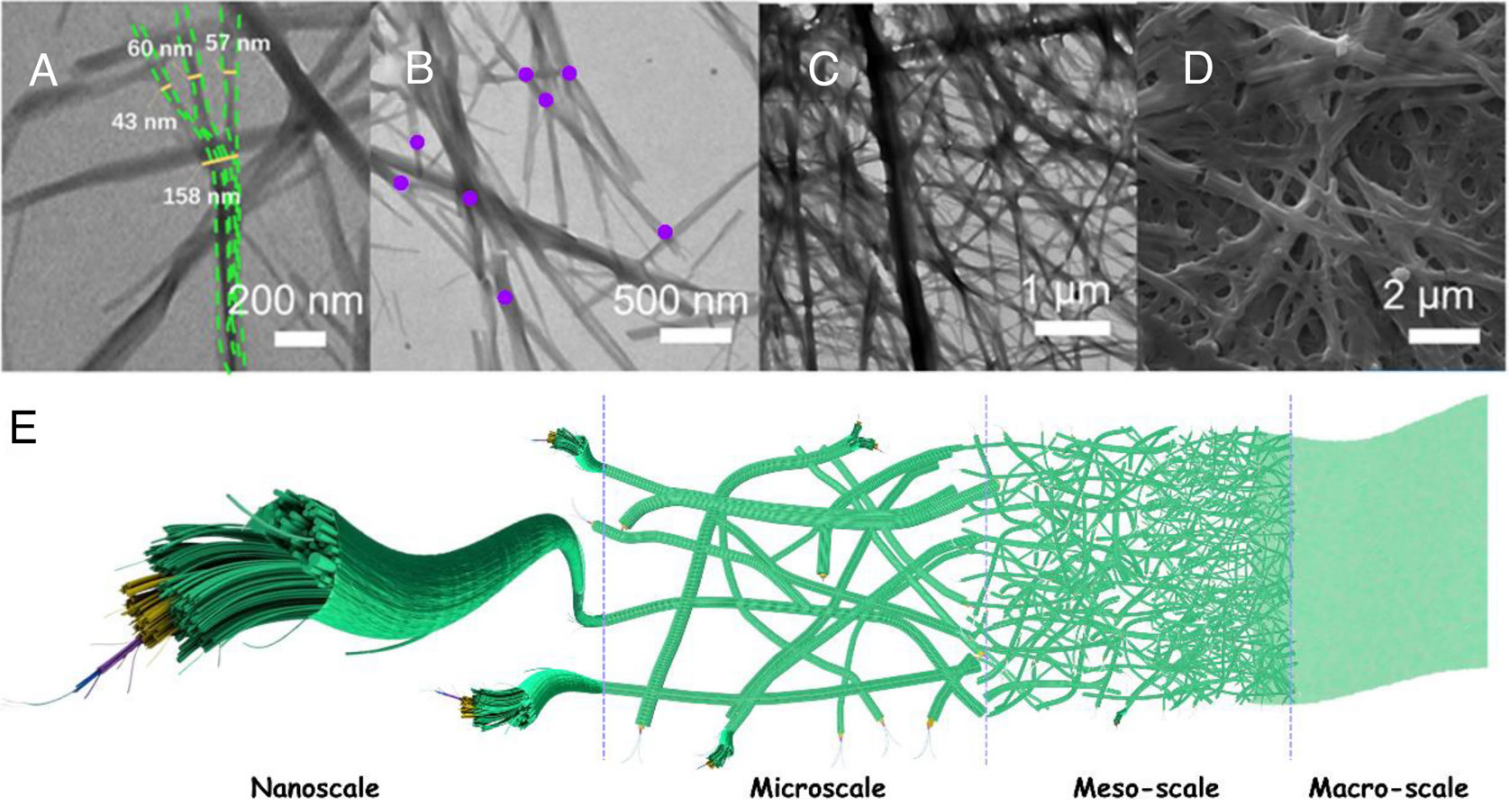
Transmission electron microscopy (TEM) images of (*A*) three thin fibers formed into a thicker fiber (dotted green lines). (*B*) The overlap among fibers results in the appearance of bundle nodes (purple dots). (*C*) TEM and (*D*) SEM images of nanofibers assembled networks. (*E*) Diagram of the assembly patterns at different scales.

The Pt MOCs were synthesized according to the literature (47). As shown in Fig. 1 *A* and *B*, the Pt MOCs preferentially formed nanoscale fibers at the initial stage and then tended to form thicker fibers. The dotted green lines in Fig. 1*A* show that the three thin nanofibers formed a thick fiber by partially aligned. As shown in Fig. 1*B*, the overlap between fibers results in the appearance of nodes (purple dots), which further assemble to form networks at a large scale (Fig. 1 *C* and *D* and *SI Appendix*, Fig. S1). The assembly patterns at different scales are displayed in Fig. 1*E*. Initially, the MOC-based nanofibers close to form thicker fibers, then close to form nodes and resulted in the appearance of networks; finally, thicker and larger networks on a large scale are formed by the overlap of these bundle networks, ultimately generating a centimeter-scale film.

**Fig. 2.**
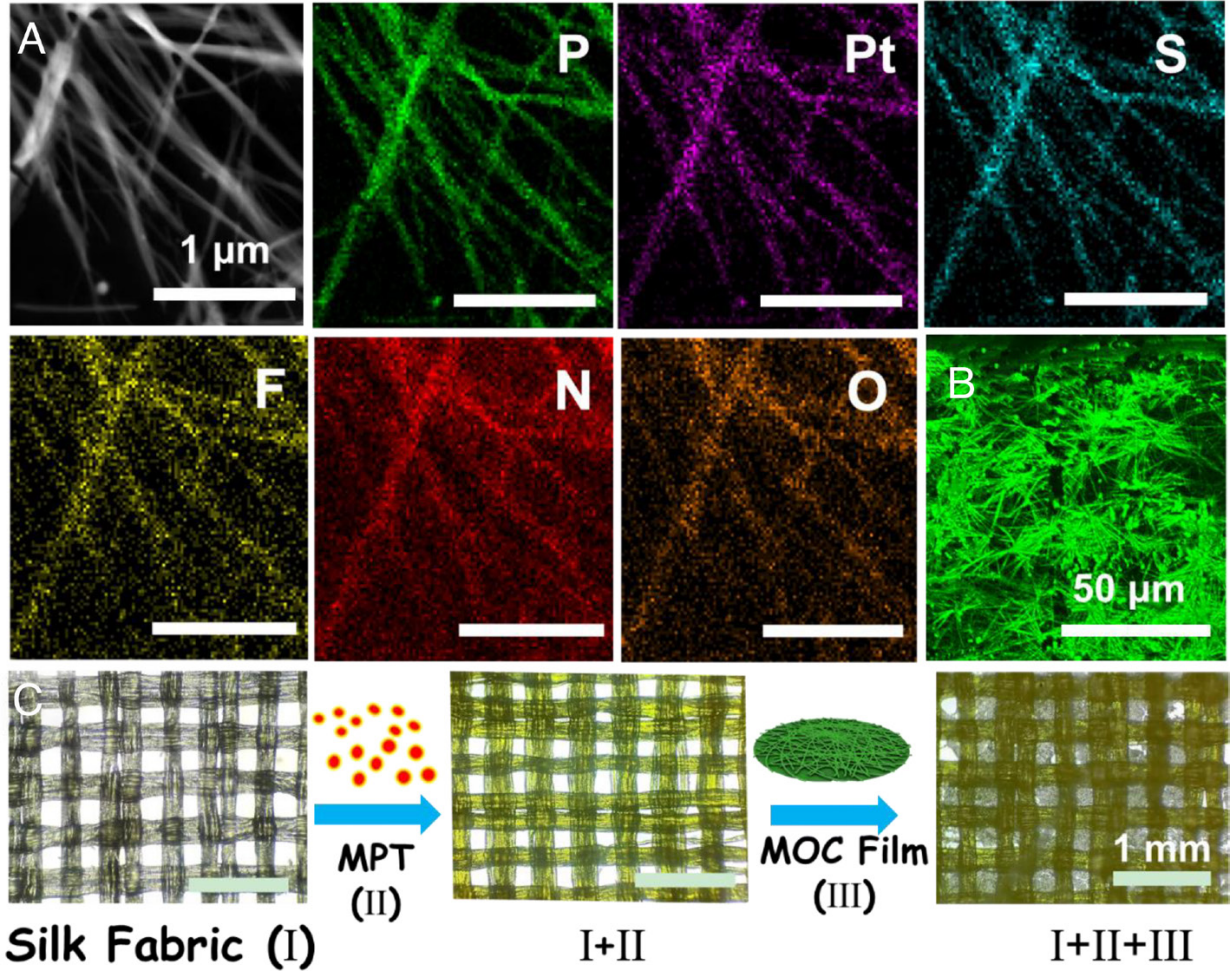
(*A*) EDX mapping of the centimeter-scale film. (*B*) CLSM image of the centimeter-scale film. (*C*) Bright-field microscopy images of the formation process of complex I+II+III.

The elemental distribution [determined by energy-dispersive X-ray spectroscopy (EDX)] and confocal laser scanning microscopy (CLSM) results provided additional evidence that the network was formed by Pt MOCs (Fig. 2 *A* and *B*). As shown in Fig. 2*C* and *SI Appendix*, Figs. S2–S5, a wound dressing (I+II+III) was prepared by the first staining of MPT(II) on a silk fabric (I) and then coating it with a Pt MOC film (III). Thereafter, the Pt MOC film was observed on the silk fabric substrate and displayed bright fluorescence under UV light (*SI Appendix*, Fig. S4). The stability of the I+II+III complex in aqueous solution was confirmed via UV‒Vis absorption and fluorescence spectroscopic analyses, and no obvious changes were observed after incubation in PBS for 5 h (*SI Appendix*, Figs. S6 and S7).

**Fig. 3.**
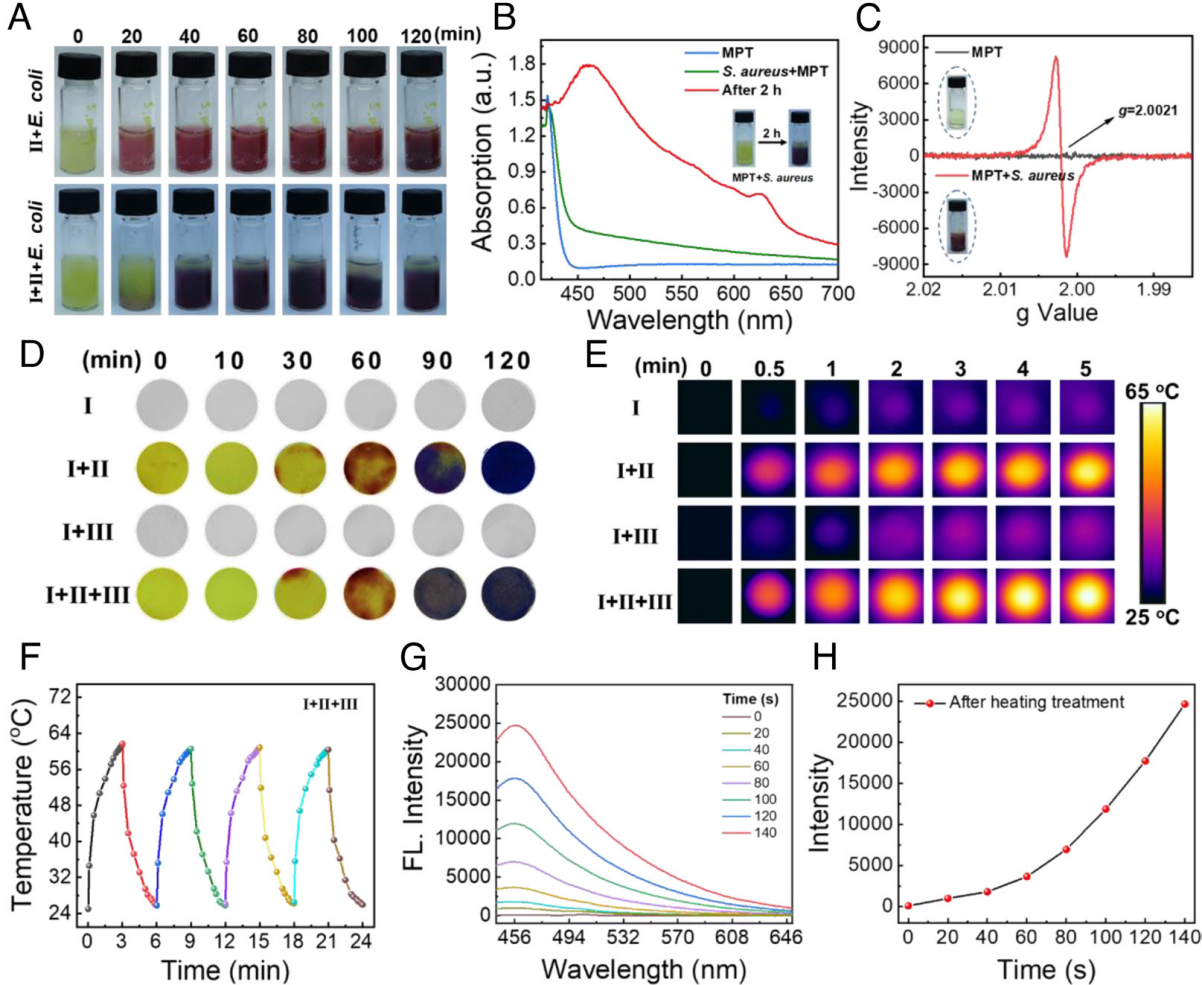
(*A*) Photographs of the changes in color of complex II and I+II after cocultivation with *S. aureus* solution (1 × 10^8^ CFU mL^−1^). (*B*) UV‒Vis spectra and (*C*) EPR spectra of MPTs incubated with *S. aureus* solution (1 × 10^8^ CFU mL^−1^) at 37 °C for 2 h. (*D*) Photographs of the samples with color changes for real-time bacterial monitoring after incubation with *S. aureus* solution (1 × 10^8^ CFU mL^−1^) on the plate. (*E*) Photothermal images of the samples under light irradiation (660 nm laser, 0.5 W/cm^2^) after incubation with *S. aureus* solution (1 × 10^8^ CFU mL^−1^) at 37 °C for 2 h on the plate. (*F*) Photothermal recycling of complex I+II+III after on-off light irradiation cycles (660 nm laser, 0.5 W/cm^2^) measured with *S. aureus* solution (1 × 10^8^ CFU mL^−1^, basic approach: First, complex I+II+III was immersed in the bacterial solution. Then, the color change of complex I+II+III could be observed. The temperature changes during on–off light irradiation cycles (660 nm laser, 0.5 W/cm^2^) in *S. aureus* solution were recorded using a thermal imaging camera to obtain the photothermal cycles curve. (*G*) Fluorescence changes in the supernatant of complex I+II+III during heating at 55 °C. (*H*) Intensity of the emission peak at 451 nm versus time.

To determine why the MPT(II)-stained silk fabric changed color in the presence of bacteria, complex II and I+II were cocultured with *Staphylococcus aureus* (*S. aureus*) and *Escherichia coli* (*E. coli*) (Fig. 3*A*). As shown in the *SI Appendix*, Figs. S8 and S9, compared with MPT alone, MPT stained on the silk fabric changed color from light yellow to deep purple after cocultivation with bacteria. However, the groups without MPT did not display color changes, indicating that the observed color changes were related to the mixture of MPT solution with bacteria. Furthermore, to investigate whether MPT reduction to the MPT radical in the presence of bacteria was a reversible process, color changes of PBS and I+II after repeated addition and removal of the *S. aureus* solution (1.0 × 10^8^ CFU mL^−1^) were studied. After the removal of the *S. aureus* solution, the color of complex I+II returned to normal after 1 h. Upon reintroduction of the *S. aureus* solution and cocultivation at 37 °C, the color of complex I+II turned dark purple again. This phenomenon was consistent upon repetitive operation, indicating MPT reduction to the MPT radical in the presence of bacteria was a reversible process, thus providing real-time monitoring of bacterial infection (*SI Appendix*, Fig. S10). Due to the transmembrane redox potential of the final stage of bacterial respiration, bacteria exhibit a certain reduction ability, and we hypothesize that this reduction may contribute to the generation of MPT radicals. To prove this assumption, UV‒Vis absorption spectroscopy was applied to study the changes in MPT after incubation with bacteria. The results showed a new absorption peak at approximately 630 nm, which was attributed to the characteristic absorption of MPT radicals (Fig. 3*B* and *SI Appendix*, Fig. S11). As shown in Fig. 3*C* and *SI Appendix*, Fig. S12, the generation of MPT radicals was further confirmed by electron paramagnetic resonance (EPR), and an EPR signal with a *g* factor of 2.0021 was observed after cocultivation with bacteria. These results indicate that the changes in the color of the MPT-stained silk fabric were caused by the in situ bacterial reduction of MPT to its radical form.

Interestingly, the generated MPT radical exhibited a notable photothermal effect under 660 nm light irradiation (*SI Appendix*, Figs. S13–S15); the temperature increased significantly to 55 °C, and the photothermal conversion efficiency (η) values were calculated to be 28.26% (for *E. coli*) and 30.11% (for *S. aureus*) (*SI Appendix*, Fig. S16). Encouraged by these color changes and remarkable photothermal effects, complex I+II+III was anticipated to serve as a wound dressing with bacterial sensing and wound healing properties. Thus, complexes I+II+III were cut into circular patches with a diameter of 1 cm and cocultured with bacteria. As shown in Fig. 3*D*, color changes were observed in the complex I+II and I+II+III groups after cocultivation with bacteria, indicating the feasibility of in situ bacterial monitoring of complex I+II. We then explored the in situ photothermal effect of complex I+II+III after the color change, and the results showed that the temperature could be rapidly increased to 63 °C within 3 min (Fig. 3*E* and *SI Appendix*, Figs. S17–S19). In addition, after multiple on/off 660 nm laser irradiation cycles, the maximum temperature exhibited no significant changes, indicating the photothermal stability of the wound dressing (Fig. 3*F*). Due to the convenient photothermal effect of complex I+II+III, we suspected that the Pt MOC film would disassemble under thermal stimulation because of the intensification of the molecular motion. Thereafter, the disassembly behavior of the Pt MOC film was investigated through heating at 55 °C, and the behavior was monitored by measuring the changes in the fluorescence of the Pt MOCs in the supernatant solution. As shown in Fig. 3 *G* and *H*, the fluorescence intensity increased with heating time, indicating the disassembly and release of Pt MOCs (*SI Appendix*, Fig. S20). To further investigate the integrity of the Pt MOCs after thermally induced release, the NMR spectra of the Pt MOCs before and after heating were examined. No significant changes were observed in the spectra, indicating that thermal treatment could only induce the disassembly of the Pt MOC film suprastructure, while the Pt MOCs themselves remained intact after thermally induced release (*SI Appendix*, Fig. S21).

**Fig. 4.**
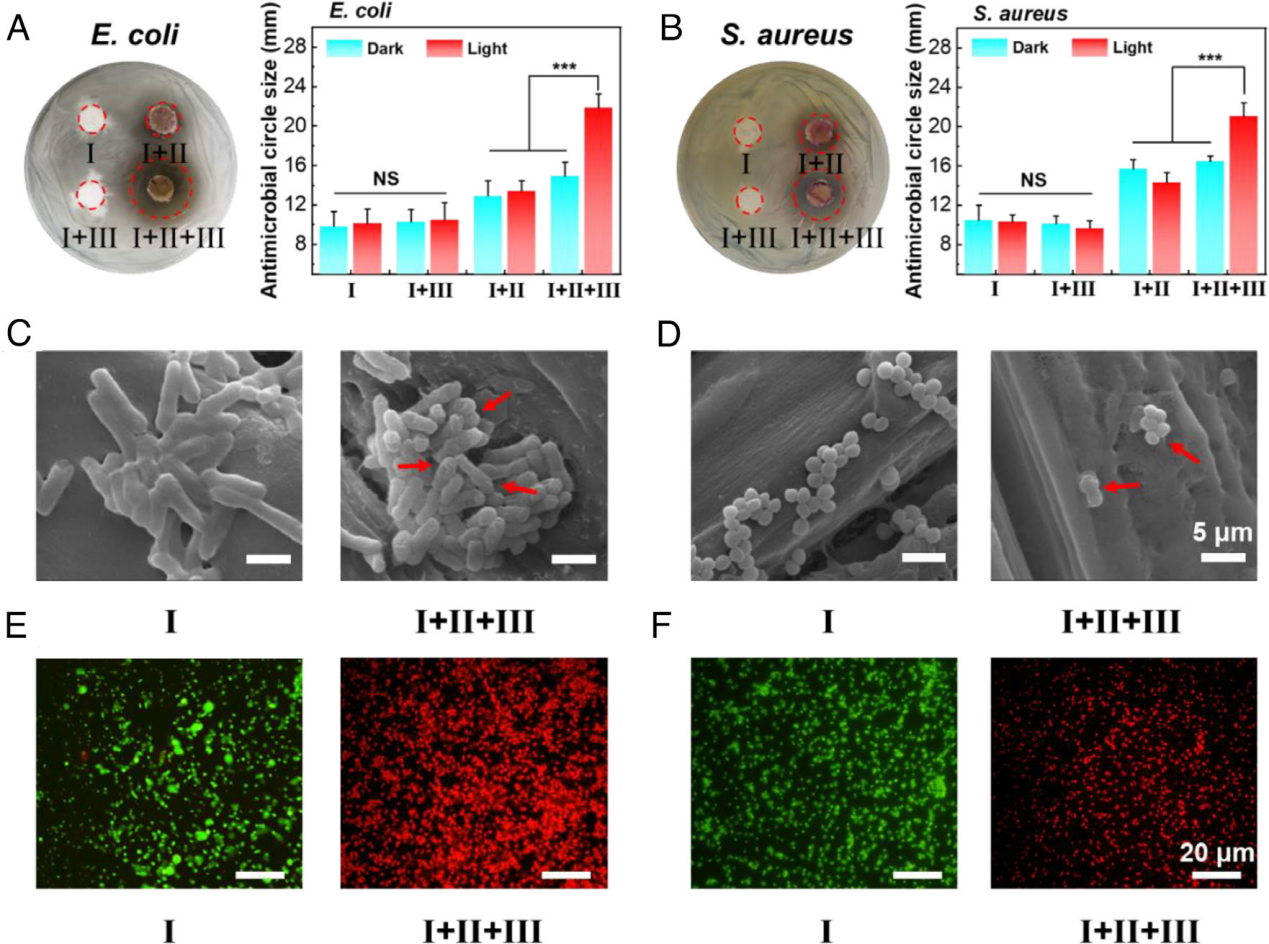
Antibacterial effects of complex I+II+III against (*A*) *E. coli* and (*B*) *S. aureus*. Disk diffusion assay results for different treatments and histogram representation of antibacterial sensitivity under light (660 nm laser, 0.5 W/cm^2^, 5 min) and dark conditions, ***P < 0.001). Corresponding SEM images of (*C*) *E. coli* and (*D*) *S. aureus* after complex I and I+II+III treatments. Corresponding confocal fluorescence images of (*E*) *E. coli* and (*F*) *S. aureus*.

In a subsequent experiment, the spread-plate disc diffusion method was used to investigate the antibacterial ability of complex I+II+III. As shown in Fig. 4 *A* and *B* and *SI Appendix*, Fig. S22, according to the sizes of the inhibition zones, there were significant differences in the antibacterial effects among the four samples under the same conditions. The I+II dark/light and I+II+III dark groups had the same antibacterial activity due to the infiltration of a small amount of MPT from complex I+II+III into Luria–Bertani agar plates. Notably, the I+III dark/light groups and I+II+III dark groups exhibited no obvious antibacterial activity, indicating that the Pt MOC film exhibited low toxicity to bacteria. Controlling the toxicity and side effects of individual Pt MOCs is difficult, whereas, in the formed centimeter-scale Pt MOC film, Pt MOCs with antibacterial effects were encapsulated inside the film, further decreasing the contact between the Pt MOCs and bacteria, thus making them nonantibacterial. In addition, the strongest antibacterial effect was observed after the I+II+III light treatment, which may be caused by the photothermal-induced disassembly of the Pt MOC film; additionally, the general polymer membrane had no antibacterial activity (*SI Appendix*, Fig. S23).

Thereafter, the changes in bacterial morphology were investigated by SEM (scanning electron microscopy) imaging (Fig. 4 *C* and *D* and *SI Appendix*, Fig. S24). The bacteria had intact and smooth surfaces and no obvious morphological changes after the I/I+III dark/light treatments, while their surface became rough and crumpled after the I+II/I+II+III dark/light treatments. The bacteria showed much more severe damage after I+II+III light treatment. The above results were further confirmed by a live/dead staining assay. As shown in Fig. 4 *E* and *F* and *SI Appendix*, Fig. S25, red fluorescence appeared after the I+II and I+II+III light treatments; the strongest red fluorescence was observed after the I+II+III light treatment, reflecting the synergistic eradication effect of *E. coli* and *S. aureus*.

**Fig. 5.**
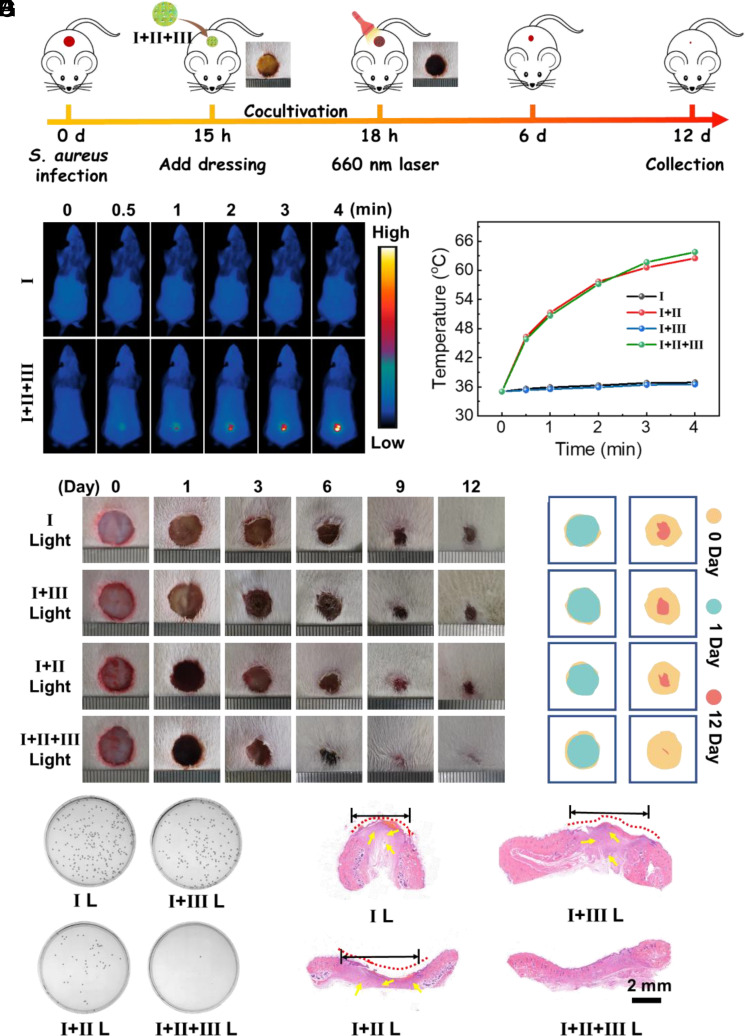
(*A*) Schematic diagram of the *S. aureus* infection and complex I+II+III treatment process in vivo. (*B*) Infrared thermal images of the wound sites of rats and (*C*) the corresponding temperature curves under 660 nm light irradiation (0.5 W/cm^2^). (*D*) Photographs of *S. aureus*-infected wounds treated with different samples from day 0 to day 12. (*E*) Schematic images of wound contraction after different treatments on day 0 and day 12. (*F*) Photographs of the bacterial colonies on agar plates from the wound sites on the first day after different treatments (n = 3). (*G*) H&E staining images of healed skin tissues after different treatments.

In view of the remarkable synergistic antibacterial effect in vitro, the antibacterial potential of complex I+II+III was evaluated in vivo (Fig. 5*A*). To investigate the influence of photothermal heating on normal tissue, the photothermal effect of complex I+II+III on normal skin tissue was investigated. As shown in *SI Appendix*, Fig. S26, complex I+II+III had no adverse effects on skin tissue after short-term laser irradiation, indicating that the thermal damage to the cells was acceptable. Circular wounds were created on the backs of 3-wk-old female Sprague‒Dawley rats and *S. aureus* solution was added to the wounds to establish infection models (50 μL, 1.0 × 10^8^ CFU mL^−1^). After the complex materials were attached to the backs of the rat skin wounds for 3 h, color changes were observed in the I+II and I+II+III groups. As shown in Fig. 5 *B* and *C*, after 660 nm light irradiation for 3 min (0.5 W/cm^2^), the temperature of the wounds treated with the I+II/I+II+III light treatments rapidly increased to approximately 63 °C, while no obvious changes were observed in the other groups (*SI Appendix*, Fig. S27). Thereafter, wound healing in the rats was monitored, as shown in Fig. 5*D* and *SI Appendix*, Fig. S28. The corresponding images and calculations of wound contraction are shown in Fig. 5*E* and *SI Appendix*, Figs. S29–S31. On day 12, in contrast to those in the other groups, the wounds in the I+II+III light treatment group had the smallest wounds (0.97%), and new epidermal and smooth dermal tissues had been generated. To provide a positive control for comparisons of the results with those of the I+II+III light treatment, a previously reported silver-loaded hydrogel (CPA2 Gel) wound dressing was selected as a supplement to the relevant experiments (48). As shown in *SI Appendix*, Fig. S32, the healing effect of the infected wound after I+II+III light treatment was similar to that of CPA2 Gel after 808 nm laser irradiation (1.0 W/cm^2^, 5 min), further demonstrating that the I+II+III light group exhibited the best wound healing efficacy among the treatment groups. The plate counting method was subsequently applied to evaluate the effect of the various wound treatments in each group (Fig. 5*F* and *SI Appendix*, Figs. S33–S35). Compared with the other groups, the I+II+III light and CPA2 light groups had almost no bacteria on the agar plates, which was consistent with the in vitro antibacterial observation.

As shown in *SI Appendix*, Fig. S36, the greatest morphological change in *S. aureus* occurred after the I+II+III light treatment. These results indicated that the I+II+III light treatment had the optimal antibacterial effect. As shown in Fig. 5*G* and *SI Appendix*, Figs. S37 and S38, hematoxylin-eosin (H&E) and Masson's trichrome staining were carried out to further evaluate the histological changes in the skin tissue. On day 12, the infected wounds treated with I+II+III light exhibited nearly complete healing compared with the other groups. Furthermore, higher magnification H&E images revealed the presence of inflammatory signals (yellow arrows), such as neutrophils, in the skin tissue of the control groups. After treatment with I+II dark/light or I+II+III dark, there was some reduction in the inflammatory cells. In contrast, the I+II+III light group exhibited no apparent inflammation and regenerated dermal tissue with hair-like appendages was observed, indicating that the I+II+III light group inhibited the inflammation caused by the infection and promoted the regeneration of dermal tissue. Additionally, Masson’s trichrome staining confirmed that, compared with those in the other groups, collagen fibers in the I+II+III light group were more abundant. In summary, the results verified that the wounds regenerated significantly from skin tissue after I+II+III light treatment, and the antibacterial effect was markedly improved. However, complex I+II+III dressings can be peeled off quickly, which reduces the risk of secondary infection after treatment. Finally, the biosafety of the antibacterial dressings was further explored in vivo. As shown in *SI Appendix*, Fig. S39, the rats in each group gradually gained weight, and no obvious pathological abnormalities or damage was found in the major organs (*SI Appendix*, Fig. S40). These results consistently confirmed the excellent biosafety of complex I+II+III, demonstrating the great potential of this material for clinical translation.

## Conclusion

In summary, we successfully developed a unique wound dressing, I+II+III, constructed from silk fabric, MPT, and centimeter-thick Pt MOC film. The silk fabric acts as a substrate for centimeter-scale Pt MOC films and MPT radicals; MPT can be reduced in situ to its radical form by bacteria, which results in color changes for real-time imaging of bacterial infection, and it also shows effective photothermal effect for PTT (photothermal therapy); the centimeter Pt MOC film exhibits photothermal-induced suprastructure disassembly behavior to release Pt MOCs. Compared with those of Pt MOCs, the highly ordered Pt MOC film suprastructure exhibited improved biosafety, while it also showed enhanced antibacterial efficiency after thermally induced disassembly along with PTT. The close collaboration of I+II+III provides real-time wound infection information for timely treatment through a noninvasive technique. This work may inspire the construction and application of centimeter-scale MOC-derived suprastructures with unique properties and functions.

## Supplementary Material

Appendix 01 (PDF)

## Data Availability

All study data are included in the article and/or *SI Appendix*.
