# Supplementary material for: Supramolecular coordination platinum metallacycle–based multilevel wound dressing for bacteria sensing and wound healing
Source: Proc Natl Acad Sci U S A. 2024 Mar 25;121(14):e2318391121. doi: 10.1073/pnas.2318391121 (PMC10998585; doi:10.1073/pnas.2318391121)
Supplement: Supplementary file 1 — Appendix 01 (PDF) [file pnas.2318391121.sapp.pdf]

PNAS

Supporting Information for

Supramolecular Coordination Platinum Metallacycle-Based Multi-Level Wound Dressing for Bacteria Sensing and Wound Healing

Wen-Zhen Li, Xiao-Qiang Wang, Ling-Ran Liu, Ju Xiao, Xin-Qiong Wang, Yu-Yuan Ye, Zi-Xin Wang, Mai-Yong Zhu, Yao Sun, Peter J. Stang, Yan Sun

* Xiao-Qiang Wang, Yao Sun, Yan Sun, Peter J. Stang

Email: wangxq@wust.edu.cn, sunyaogbasp@ccnu.edu.cn, elaine.sun@henu.edu.cn, stang@chem.utah.edu

This PDF file includes:

Supporting text
Figures S1 to S40
SI References

Materials and methods

All the chemical materials were purchased from commercial suppliers without further purifications. All types of bacteria were grown in Luria-Bertani (LB) culture medium at 37 °C for 15 h before further application. Live/Dead bacterial staining kit was obtained from Yuanye Biology Company (Shanghai, China).

The ^1H NMR spectra were recorded by an Agilent 600 MHz DD2 spectrometer. The UV-Vis spectra were measured by a SP-756P UV-Vis spectrometer. The photothermal effect was recorded by FLIR A35 FOV 25 (60 Hz) thermal imaging camera. The photothermal antibacterial experiments were carried out by a DS660-MFC-2 laser (Wavelength: 660 nm; Power density: 0.5/1.0 W/cm^2). Electronic paramagnetic resonance (EPR) measurements were performed on a Bruker A300 apparatus.

Synthesis of MPT

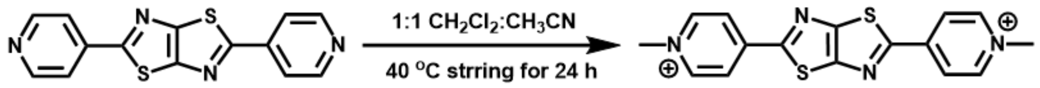

110 mg of N, N'-dipyridinium thiazolo[5,4-d]thiazole was dissolved in a mixture of 5 mL CH_2Cl_2 and 5 mL CH_3CN. Then, 100 µL of CH_3I was added, and the reaction mixture was stirred at 40 °C for 24 h. After that, the reaction mixture was centrifuged, and an orange precipitate (MPT) was obtained. The precipitate was washed with ether for 3 times and dried (1).

Fabrication of centimeter Pt MOC film

100 µL DCM solution of Pt MOCs (400 µM) was mixed with 100 µL DCM in a vial. Then, 800 µL EA was slowly added to the DCM solution, and the reaction was allowed to proceed for 48 h. For other assemblies, a similar procedure using different amounts of DCM/EA solutions was applied to tune the ratio of DCM/EA to obtain assemblies with controllable size and shape (2).

Preparation of complex I+II

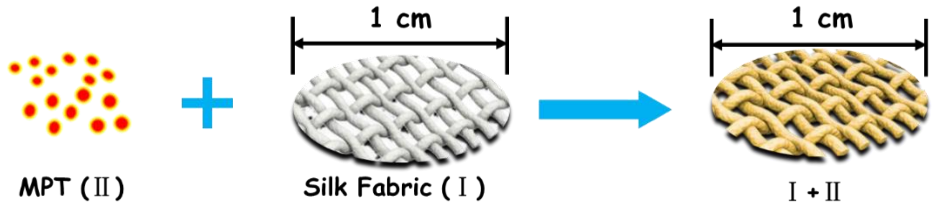

The silk fabric (I) with a diameter of approximately 1 cm was soaked in 20 mL of a 200 µM of MPT (II) solution at room temperature for 5 h. Obvious color change from white to slightly yellow was observed. Thereafter, the complex I+II was obtained and washed 3 times with H_2O and dried.

Preparation of complex I+II+III

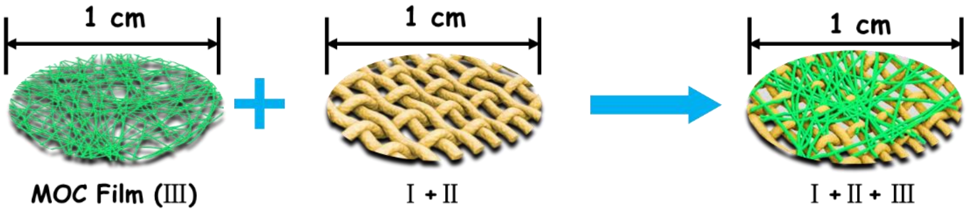

Complex I+II+III was prepared by directly transferring the centimeter Pt MOC film from DCM/EA solutions to the complex I+II (as a support substrate) with similar diameter, and dried in vacuum.

Bacteria cultivation

Gram-negative *E. coli* and gram-positive *S. aureus* were inoculated into nutrient agar by streak plate method. After being cultured in a constant temperature incubator (MJ-150-I) for 15 h, colonies

were transferred to 25 mL sterile LB medium at 37 °C and cultured to mid-log phase. After centrifugation and being washed for 3 times, bacterial suspensions with different concentrations in PBS were prepared.

Electron paramagnetic resonance (EPR) measurements

MPT (100 μM) was dissolved in PBS. Then 100 μL of the bacteria suspension (*E. coli* or *S. aureus*) was added into 3.5 mL LB medium. The medium was transferred into NMR tube and sealed. The samples were incubated at 37 °C for 12 h and then measured by EPR spectrometer (3).

In vitro photothermal performance

The 660 nm laser was produced by DS660-MFC-2 Laser Light Source, and the photothermal induced temperature change was recorded by an infrared camera. After incubation with bacteria, the solution of MPT showed obvious color change. Then the samples were irradiated by the 660 nm laser (1.0 W/cm^2) for 10 min, and obtained the temperature curve.

In vitro photothermal cycles of complex I+II+III

First, 300 μL of *S. aureus* solution (1.0×10^8 CFU mL^{-1}) was taken and added to a 12-well cell culture plate. Subsequently, a circular complex I+II+III with a diameter of 1 cm was immersed in the bacterial solution. After incubation at 37 °C for 3 h, an obvious color change from light yellow to dark purple was observed in complex I+II+III. Then, the color-changed complex I+II+III was gently transferred to a clean white foam board. Under optimal lighting condition (660 nm laser, 0.5 W/cm^2), continuous irradiation for 3 min resulted in a rapid increase in the temperature of complex I+II+III with increasing irradiation time. After 3 min, the light source was turned off, and natural cooling occurred leading to a gradual reduction in the temperature of complex I+II+III with increasing cooling time. This heating and cooling process was repeated 5 times, and the temperature changes during heating and cooling were recorded using FLIR A35 FOV 25 (60 Hz) thermal imaging camera to obtain the photothermal cycles curve as shown in Figure 3f.

In vitro antibacterial experiments of complex I+II+III

Firstly, the complex I+II+III and other control groups were transferred on the agarose culture medium plates with *S. aureus* and *E. coli*, respectively. After incubation with bacteria, the complex I+II+III showed obvious color change from slightly yellow to deep purple. Then the samples were irradiated by 600 nm light (0.5 W/cm^2) for 5 min. After that, the bacteria were further cultured in a constant temperature incubator at 37 °C for 15 h, the color change and zone of inhibition in each group was recorded by camera and analyzed.

Establishment of wound-infection model

3-week-old Female Sprague-Dawley rats were used in the animal experiments. Rat handling and procedures were guided by the Institutional Animal Care and Use Committee. Standard laboratory food and water were provided in the animal facility of Wuhan University of Science and Technology. All animal procedures were approved by the committee of the Laboratory Animal Science Department at Wuhan University of Science and Technology. Rats were anesthetized by inhalation of 1.5% isoflurane and the dorsum was shaved before surgery. One full-thickness round wound (diameter=1.0 cm) was created on the dorsum of each rat. Subsequently, 50 μL of *S. aureus* (1.0×10^8 CFU mL^{-1}) was immediately dripped onto the round wounds and evenly smeared over the wound surface. Then the wounds were fixed with elastic medical bandages. The next day, the wound-infection models were established successfully.

In vivo antibacterial effect

The wounds in each rat were treated with different samples: I Dark/Light, I+II Dark/Light, I+III Dark/Light, and I+II+III Dark/Light (0.5 W/cm^2, 4 min). To quantitatively investigate the antibacterial effect *in vivo*, the tissue fluid at day 1 was collected and homogenized in normal saline (1.0 mL). 100 μL of the diluted bacterial suspension was uniformly spread onto the fresh LB agar plates. After cultivation overnight, the bacterial colonies on the agar plates were photographed and counted.

The bacteria suspension treated by PBS with or without 660 nm laser irradiation was investigated in the control groups.

Live/Dead staining of bacteria

We studied the antibacterial properties of complex I+II+III by a bacterial live/dead staining assay. Briefly, after different treatments, the bacteria cells were co-stained by PI and FDA for 30 min in the dark, followed by washing thrice with PBS. According to the manufacturer's instructions, all bacteria were labeled by FDA and appeared green fluorescence, while dead bacteria were stained by PI and revealed red fluorescence. Finally, fluorescence images were captured using an inverted laser scanning microscopy (Olympus, FV1000).

Characterization of bacterial morphology

SEM imaging was performed to visualize the bacteria morphological changes after the antibacterial experiments. Specifically, after treatments, all collected bacteria were washed with PBS and then fixed with 2.5% glutaraldehyde solution at 4 °C for 4 h. After fixation, these specimens were serially dehydrated by graded ethanol solutions (10%, 20%, 30%, 50%, 70%, 80%, 90%, 100%). Next, the dried bacteria were sputter-coated with Au to increase conductivity, and their morphologies were observed by SEM.

Photothermal conversion efficiency

The photothermal conversion efficiencies (η) were measured according to a previously described method:(5,6)

$$\eta=[hs(T_{max}-T_{surr})-Q_{Dis}]/I(1-10^{-A}) \qquad \text{-----Equation (Se1)}$$

h is the heat transfer coefficient, s is the surface area of the container, and the value of hs is determined from the equation (Se2). Q_{Dis} represents heat dissipated from the laser mediated by the solvent and container. We chose 660 nm laser irradiation (Power density: 1.0 W/cm^2). For convenience, I is the laser power and A is the absorbance at 660 nm.

$$hs=mC/\tau_s \qquad \text{-----Equation (Se2)}$$

m is the mass of the solution containing the photoactive material, C is the specific heat capacity of the solution, and τ_s is the associated time constant, which can be determined from equation (Se3).

$$t=-\tau_s \ln(\theta) \qquad \text{-----Equation (Se3)}$$

θ is a dimensionless parameter, known as the driving force temperature, as calculated using equation (Se4).

$$\theta=(T-T_{Surr})/(T_{Max}-T_{Surr}) \qquad \text{-----Equation (Se4)}$$

T_{Max} and T_{Surr} are the maximum steady state temperature and the environmental temperature, respectively. To obtain the photothermal conversion efficiencies of complex I+II, the temperature of the MPT (0.1 mM) solution was raised by 30 °C upon irradiation for 10 min at 660 nm laser irradiation. Moreover, the water heat capacity is 4.2 J g^{-1} °C^{-1}).

Statistical analysis

All experiments were conducted at least three times unless otherwise noted. The statistical analysis was performed using OriginPro 8.0, followed by a student's t-test and one-way analysis of variance (ANOVA). *P<0.05 was considered statistically significant. **P<0.01 and ***P<0.001 were considered highly significant.

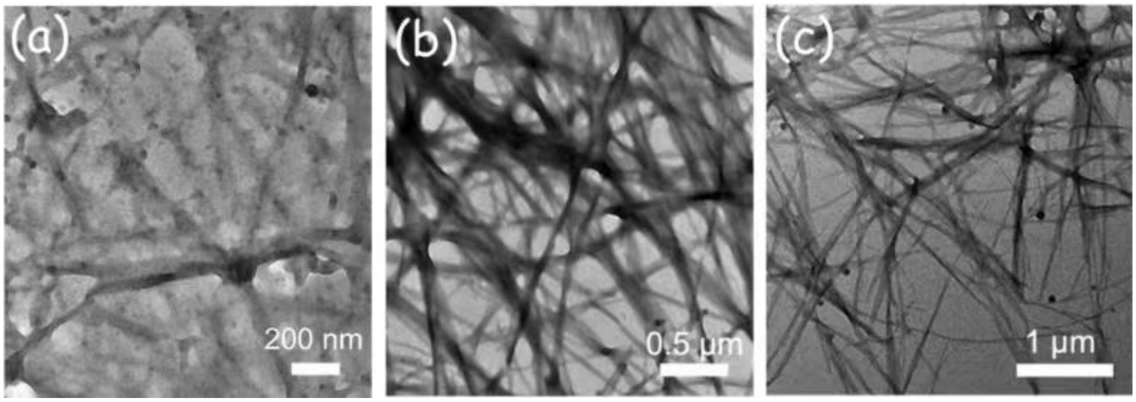

Fig. S1. TEM images of (a) elongated nanofibers generated from a nanosphere, (b-c) the fusion among fibers results in the formation of network.

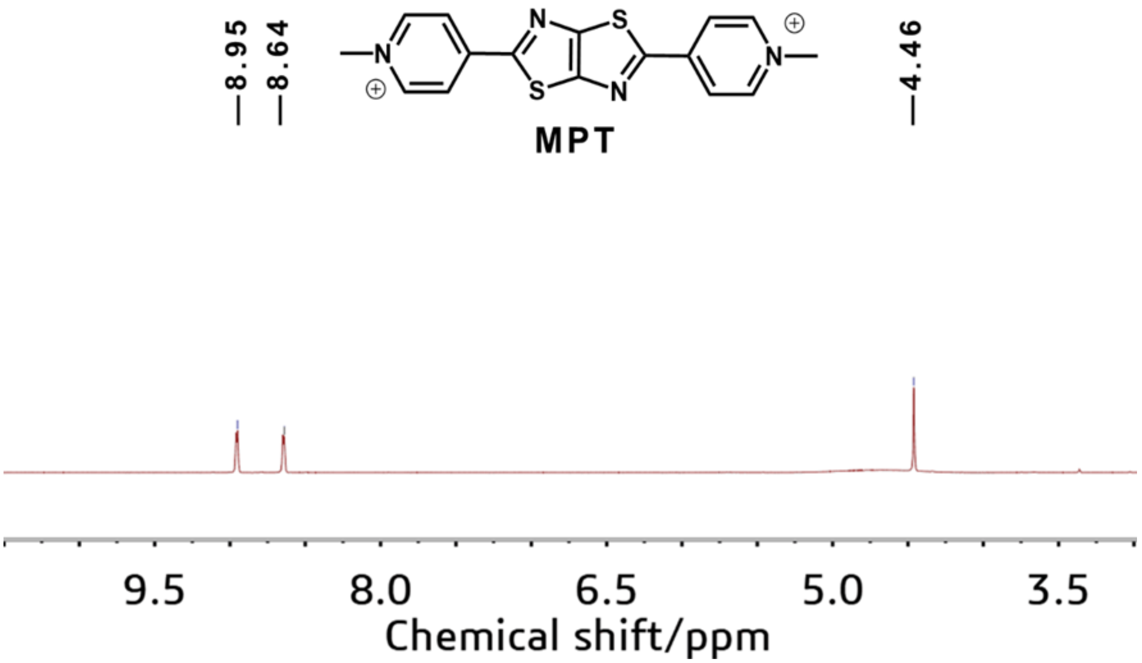

Fig. S2. ^{1}H NMR of MPT in D_2O (25 ºC, 600 MHz).

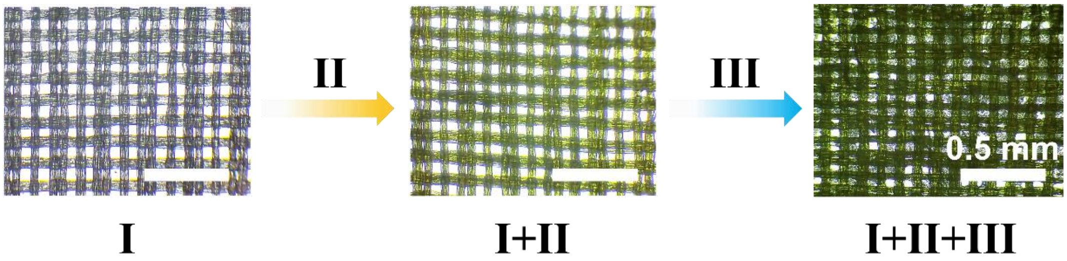

Fig. S3. Bright-field microscopy images of the formation process of complex I+II+III.

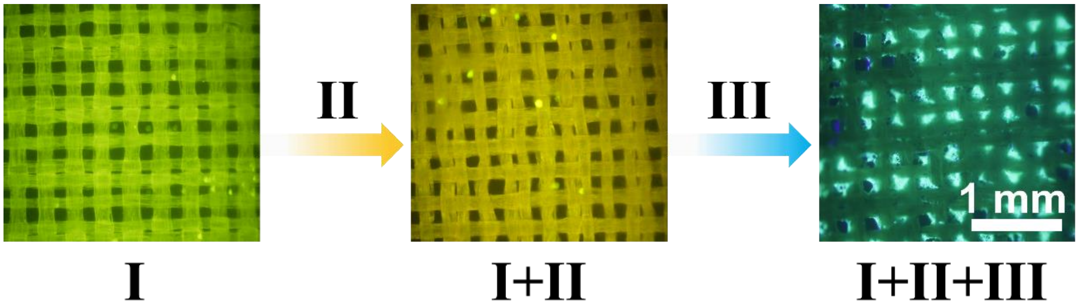

Fig. S4. The inverted fluorescent microscopy photographs of the formation process of complex I+II+III under 425 nm light.

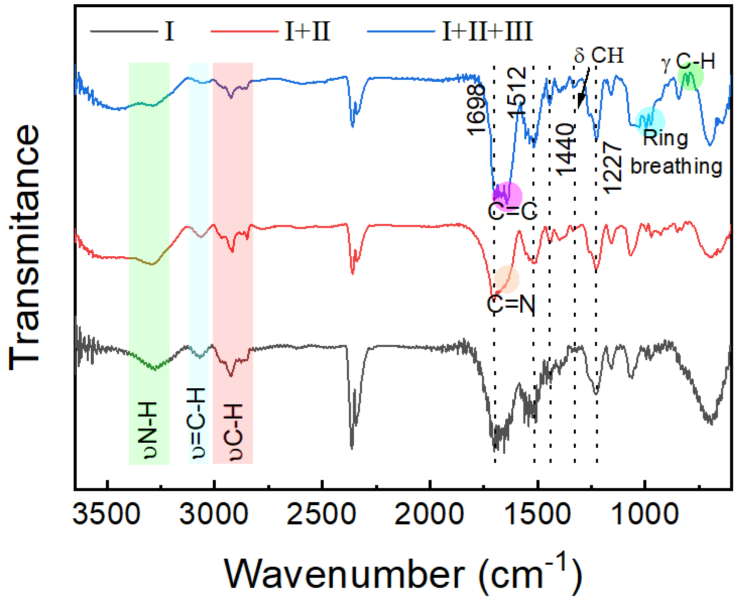

Fig. S5. Fourier transform infrared spectroscopy (FTIR) spectra of complex I, I+II and I+II+III.

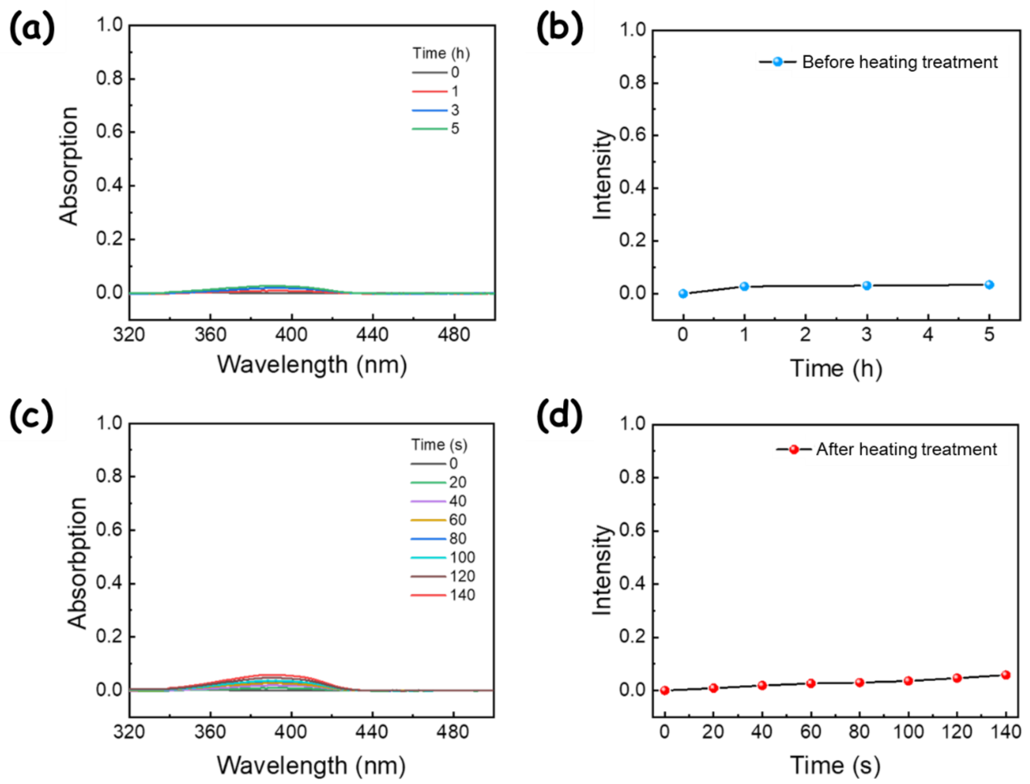

Fig. S6. (a, c) UV-Vis spectroscopic analysis of supernatant solution and (b, d) corresponding release relationship of MPT from complex I+II+III with time via different treatments.

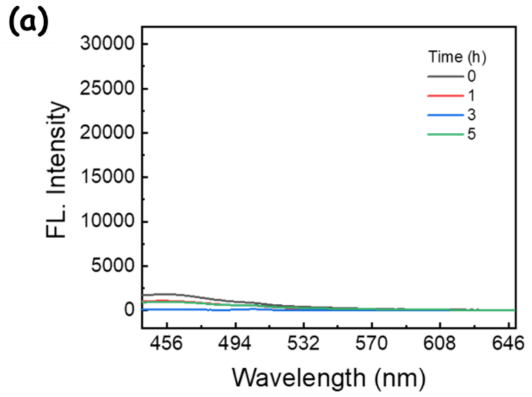

Fig. S7. (a) Fluorescence changes of supernatant solution and (b) corresponding release relationship of Pt MOCs from complex I+II+III before heating treatment.

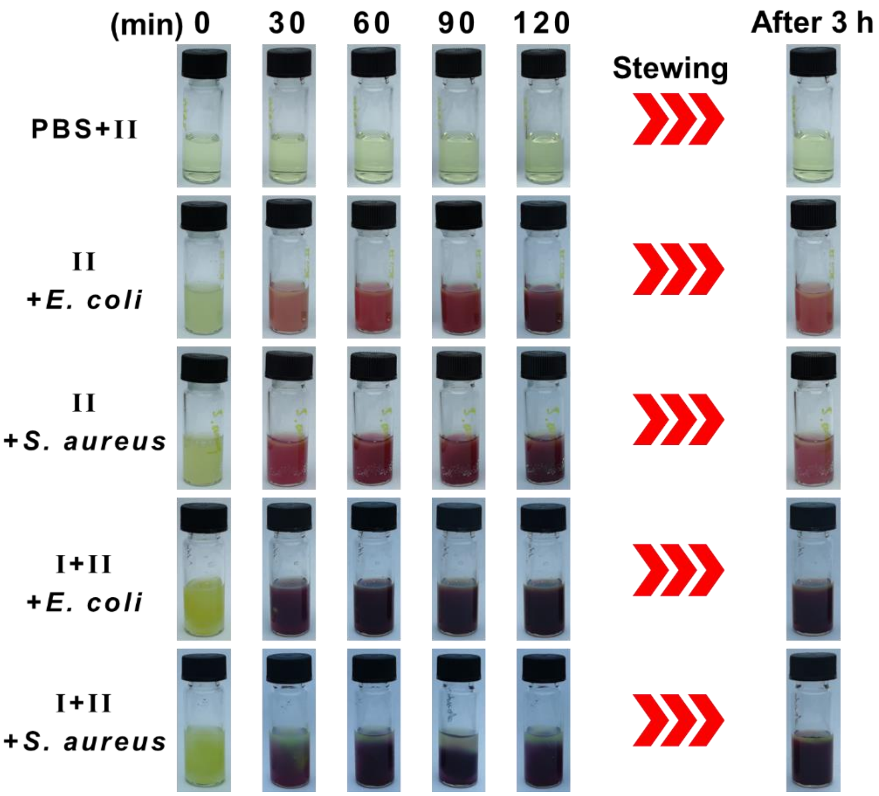

Fig. S8. Color change photographs of MPT solution cocultivation with bacteria (bacterial concentration: $1x10^8$ CFU mL^{-1}).

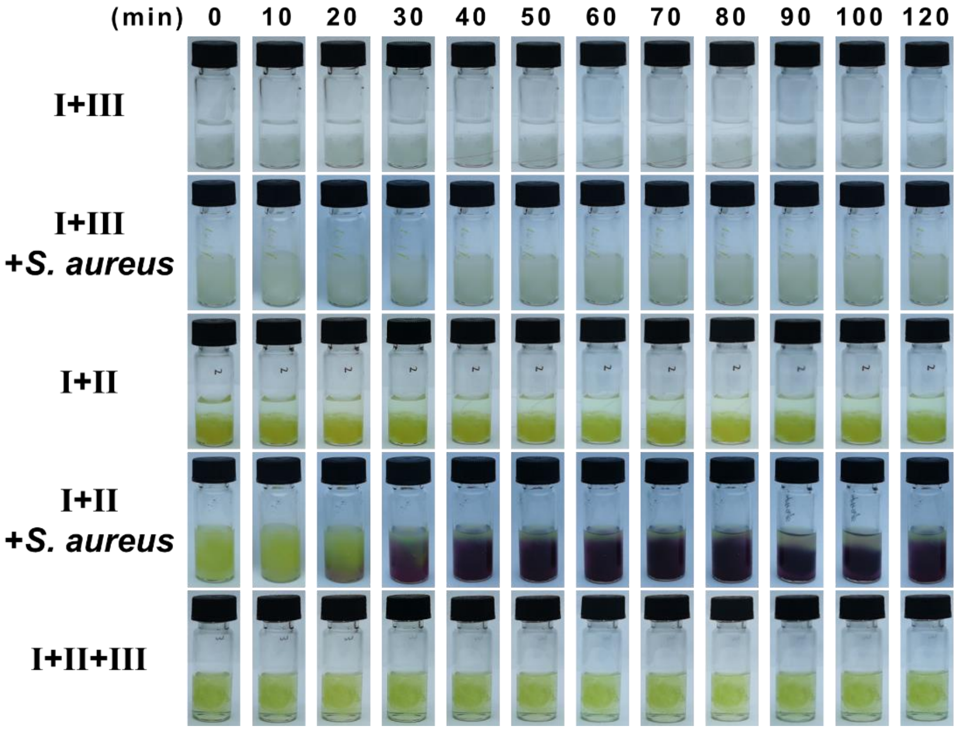

Fig. S9. Color change photographs of complex materials cocultivation with/without bacteria (Bacterial concentration: $1x10^8$ CFU mL^{-1}).

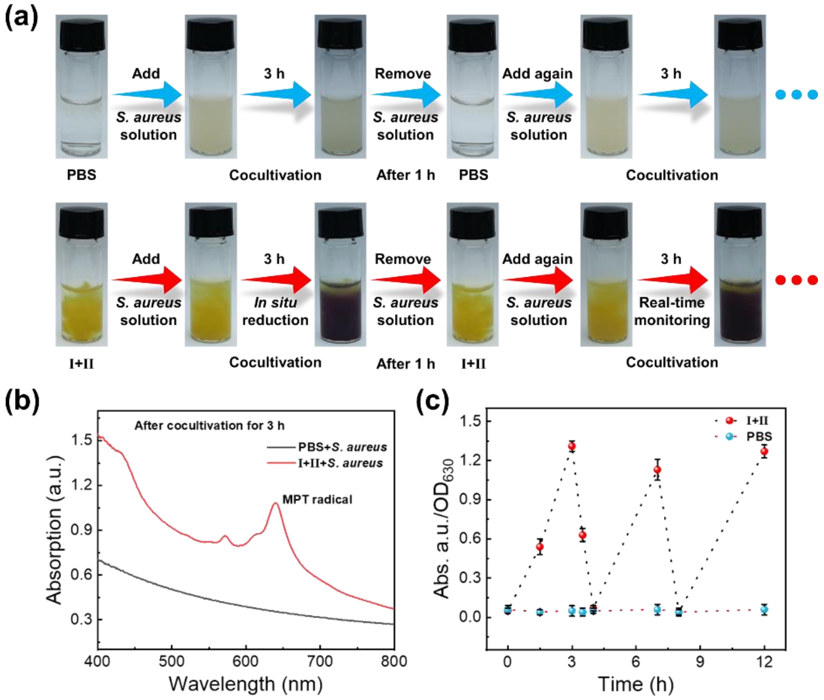

Fig. S10. (a) Color changes of PBS and I+II after repeated addition and removal of the *S. aureus* solution (1.0×10^8 CFU mL^{-1}). (b) UV-Vis spectra of PBS and I+II after cocultivation with *S. aureus* solution (1.0×10^8 CFU mL^{-1}) for 3 h. (c) Corresponding intensity of absorption peak at 630 nm with incubating time.

When adding *S. aureus* solution (1.0×10^8 CFU mL^{-1}), the intensity of absorption peak at 630 nm (OD_{630}) was measured according to a method:

$$OD_{630}=A_t-A_0 \qquad \text{-----Equation (Se5)}$$

A_t is the absorption peak intensity of PBS or I+II at 630 nm after cocultivation with *S. aureus* solution (1.0×10^8 CFU mL^{-1}), A_0 is the absorption peak intensity of the initial *S. aureus* solution at 630 nm.

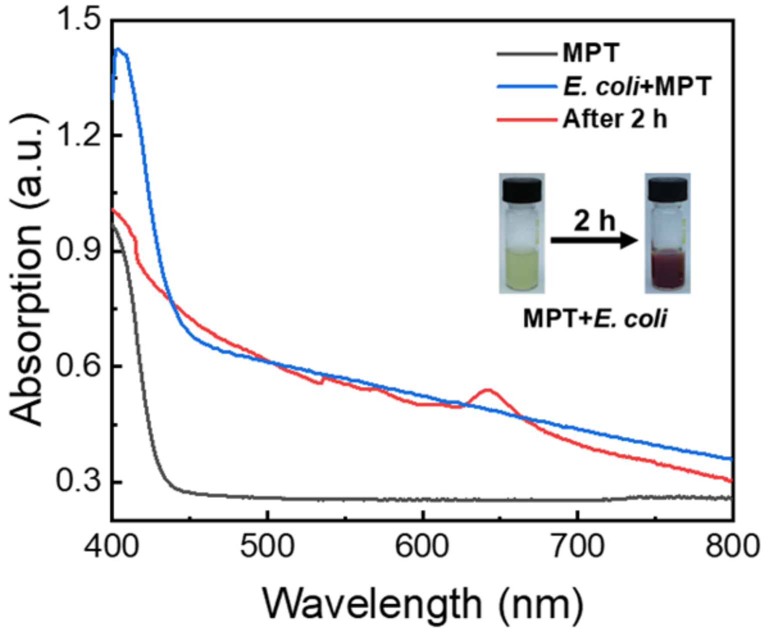

Fig. S11. UV-Vis spectra and images of MPT incubated with *E. coli* solution (1.0×10^{8} CFU mL^{-1}) at 37 °C for 2 h.

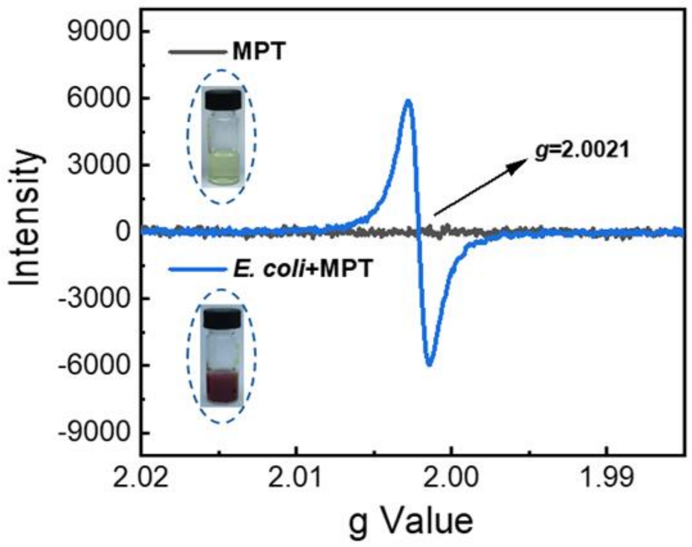

Fig. S12. Electron paramagnetic resonance (EPR) spectroscopy of MPT reduced by *E. coli* (1.0×10^8 CFU mL^{-1}).

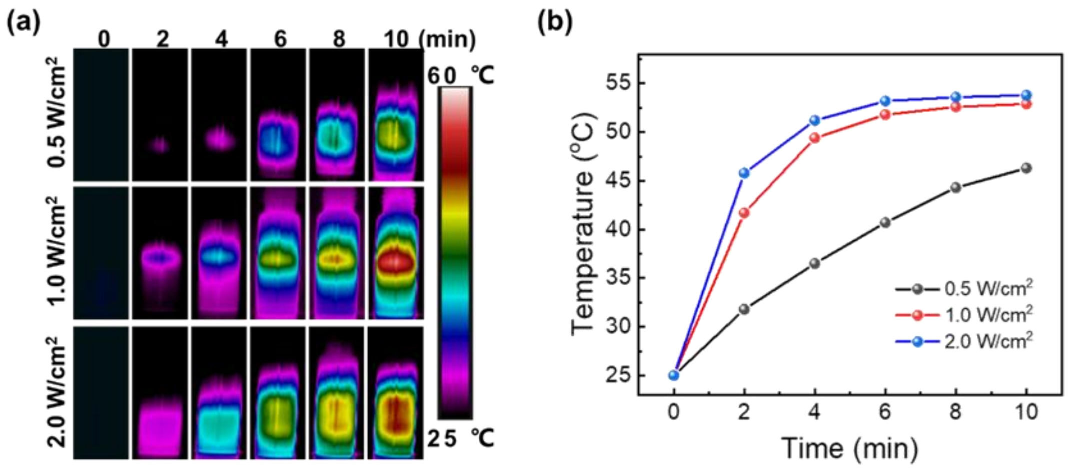

Fig. S13. Photothermal properties of complex I+II after treated by *S. aureus* solution (1.0×10^8 CFU mL^{-1}): (a) infrared thermal images and (b) photothermal temperature curves under 660 nm laser irradiation with different power densities (0.5, 1.0, and 2.0 W/cm^2).

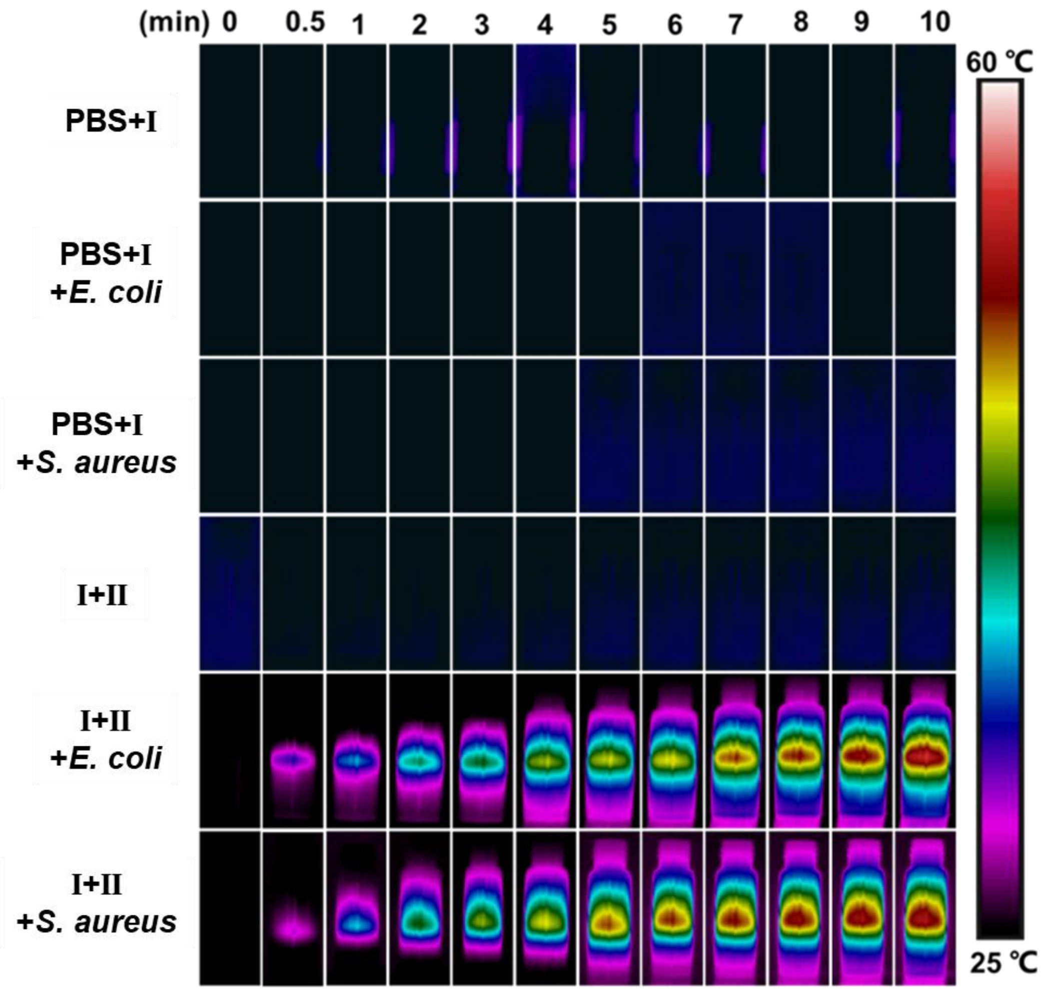

Fig. S14. Photothermal infrared images after different treatments under 660 nm laser irradiation (power density=1.0 W/cm^2).

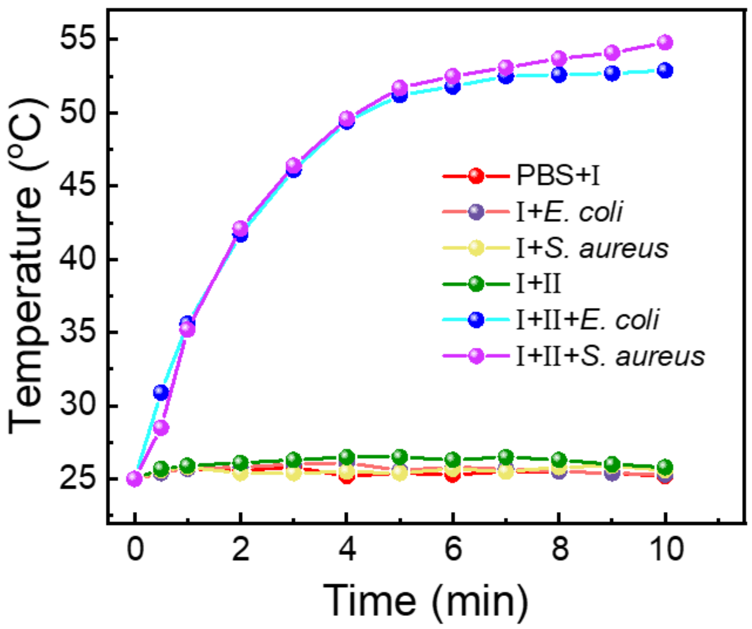

Fig. S15. Corresponding photothermal curve under 660 nm laser irradiation, (power density=1.0 W/cm^2).

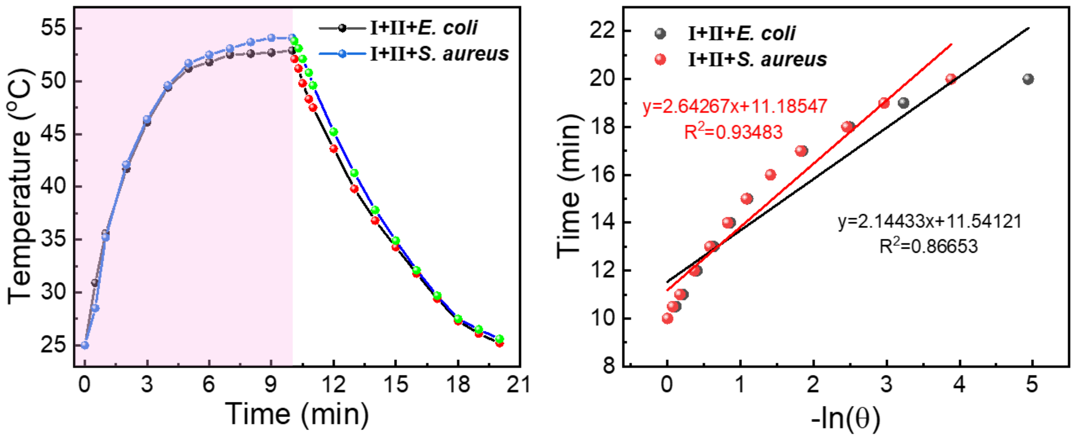

Fig. S16. Temperature change curves and the calculated time constants of I+II after cocultivation with *S. aureus* and *E. coli* (1.0×10^8 CFU mL^{-1}).

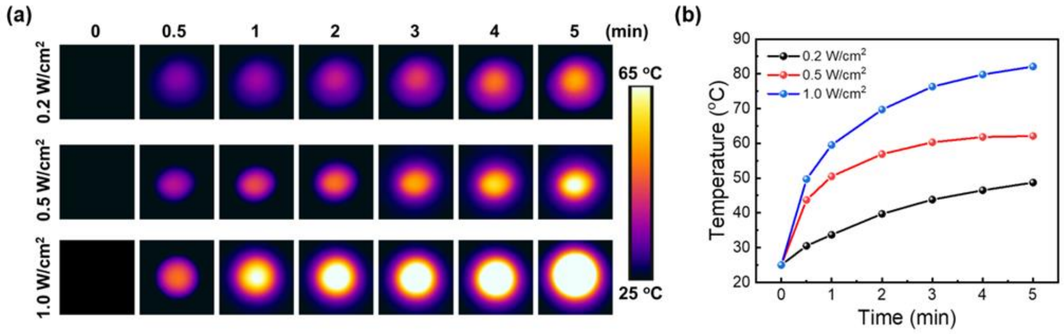

Fig. S17. Photothermal properties of complex I+II+III cocultivation with bacterial solution (1.0×10^{8} CFU mL^{-1}): (a) infrared thermal images and (b) photothermal temperature rise curves under 660 nm laser irradiation. (Power densities=0.2, 0.5, and 1.0 W/cm^2).

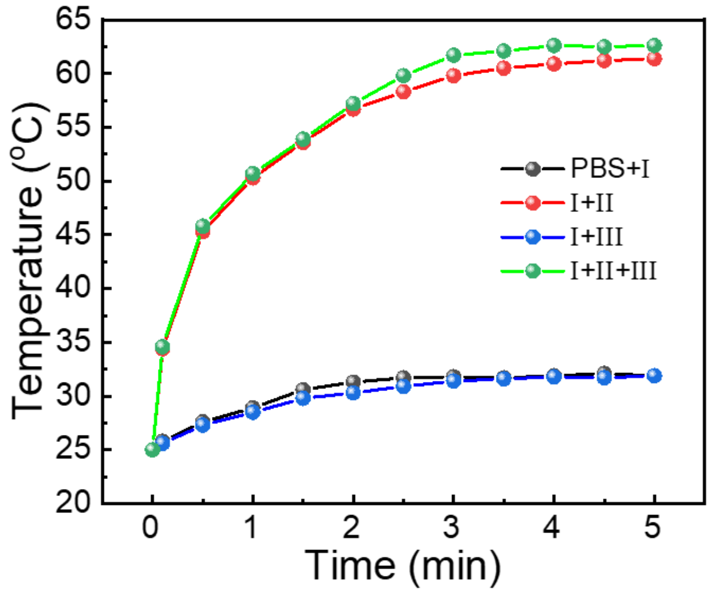

Fig. S18. The corresponding photothermal curve of the complexes (power density: 0.5 W/cm^2).

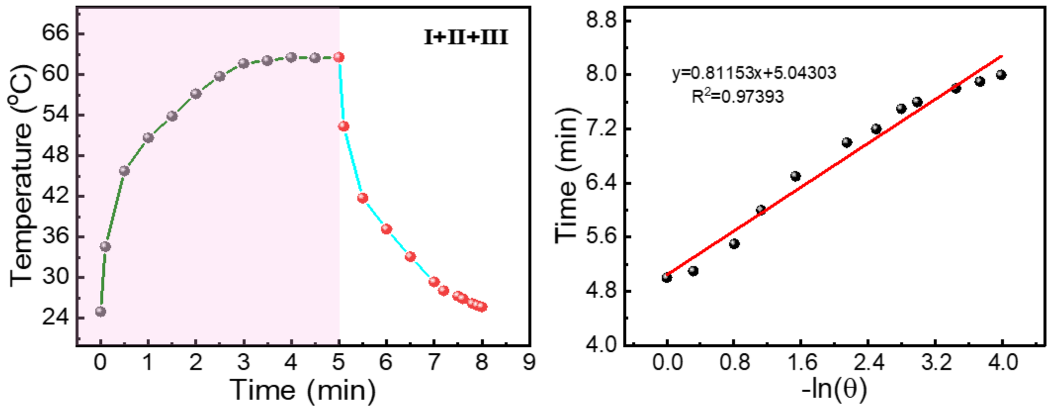

Fig. S19. Temperature change curves and the calculated time constants of complex I+II+III.

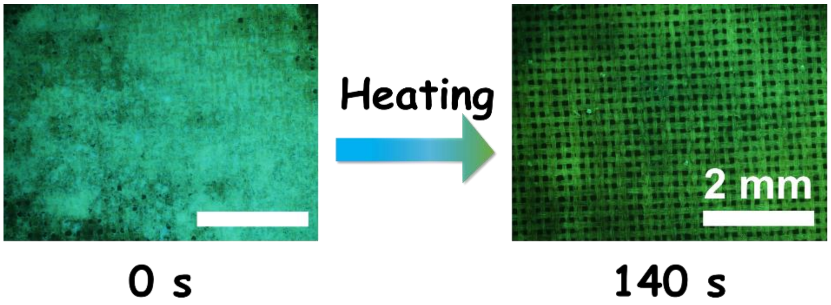

Fig. S20. The inverted fluorescent microscopy photographs of disassembly of Pt MOC film on complex I+II+III after heating treatment at 55 °C under 425 laser irradiation.

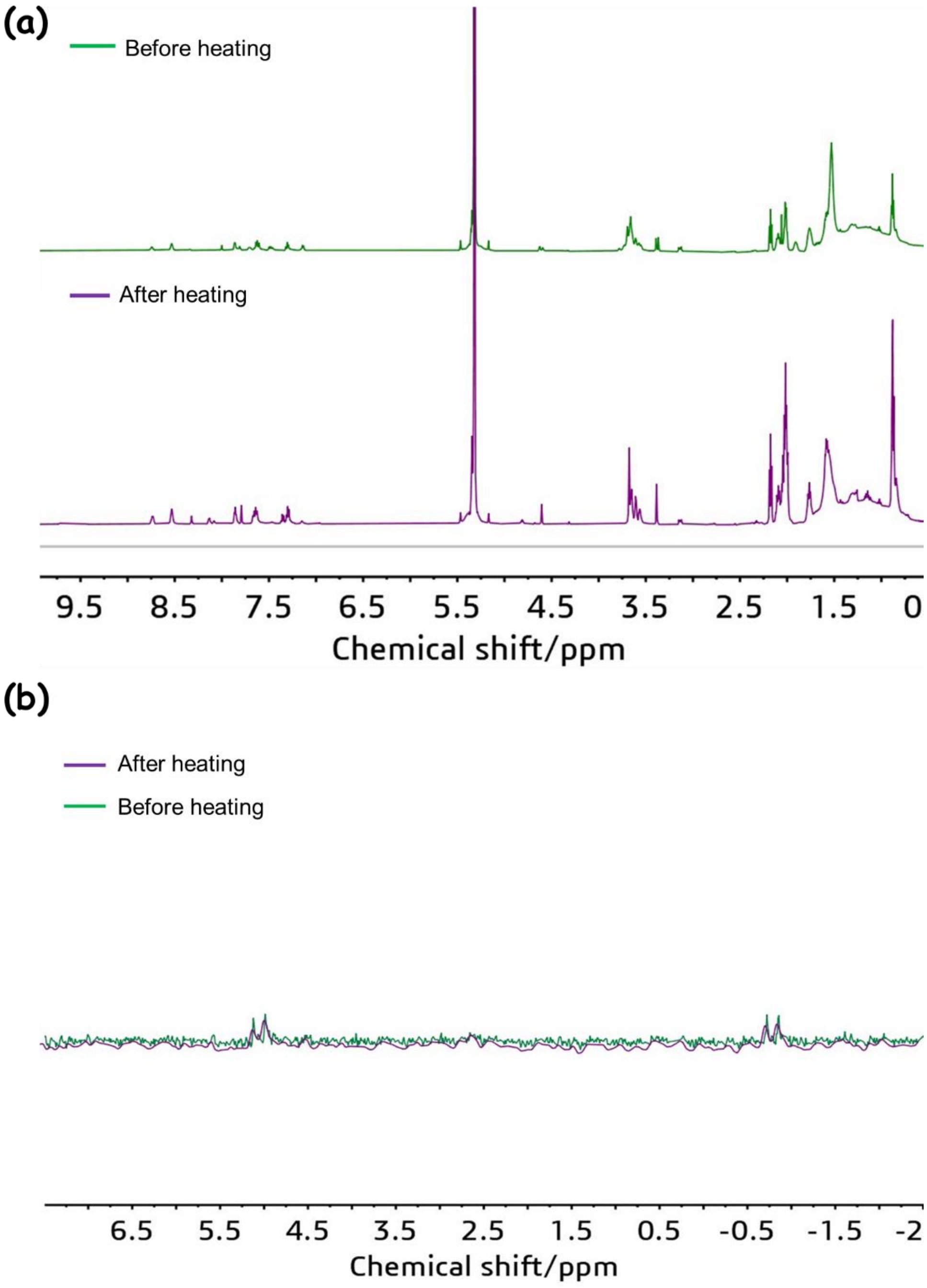

Fig. S21. (a) ^{1}H NMR spectrum and (b) ^{31}P{^{1}H} NMR spectrum of Pt MOCs (400 MHz, CD_2Cl_2) before and after heating.

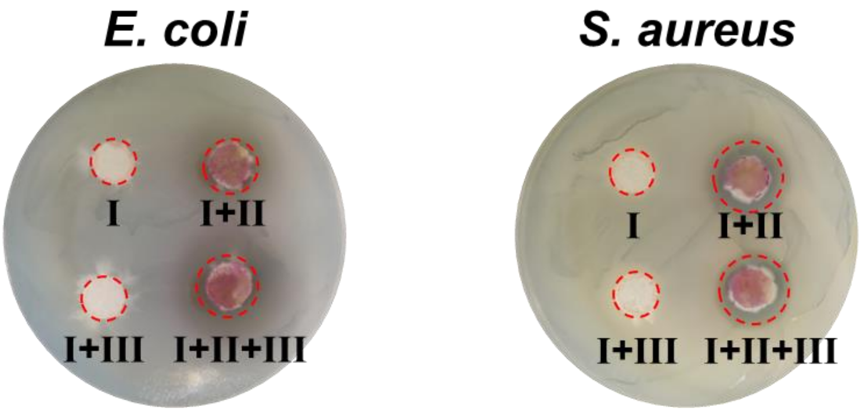

Fig. S22. Antibacterial effect of complex I+II+III against *E. coli* and *S. aureus*. Disc diffusion assay results with different treatments under dark conditions.

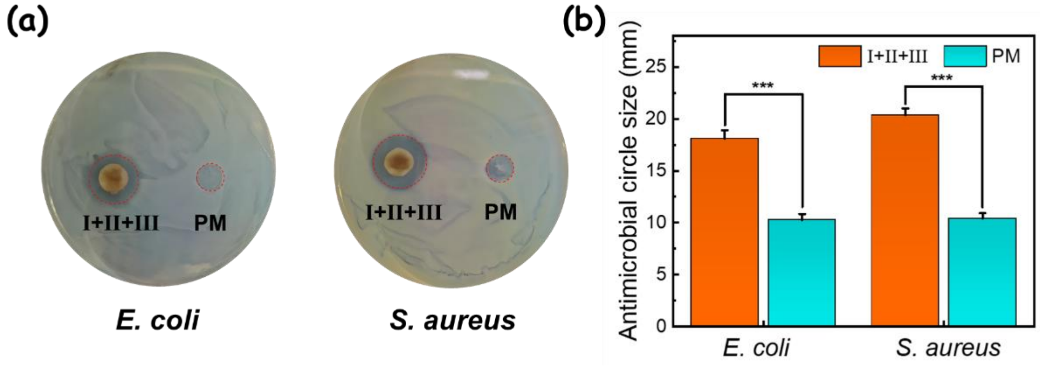

Fig. S23. (a) Antibacterial effect of complex I+II+III and polymer membrane against *E. coli* and *S. aureus*. (b) Disc diffusion assay results with different treatments under dark conditions (n=3, ***P<0.001).

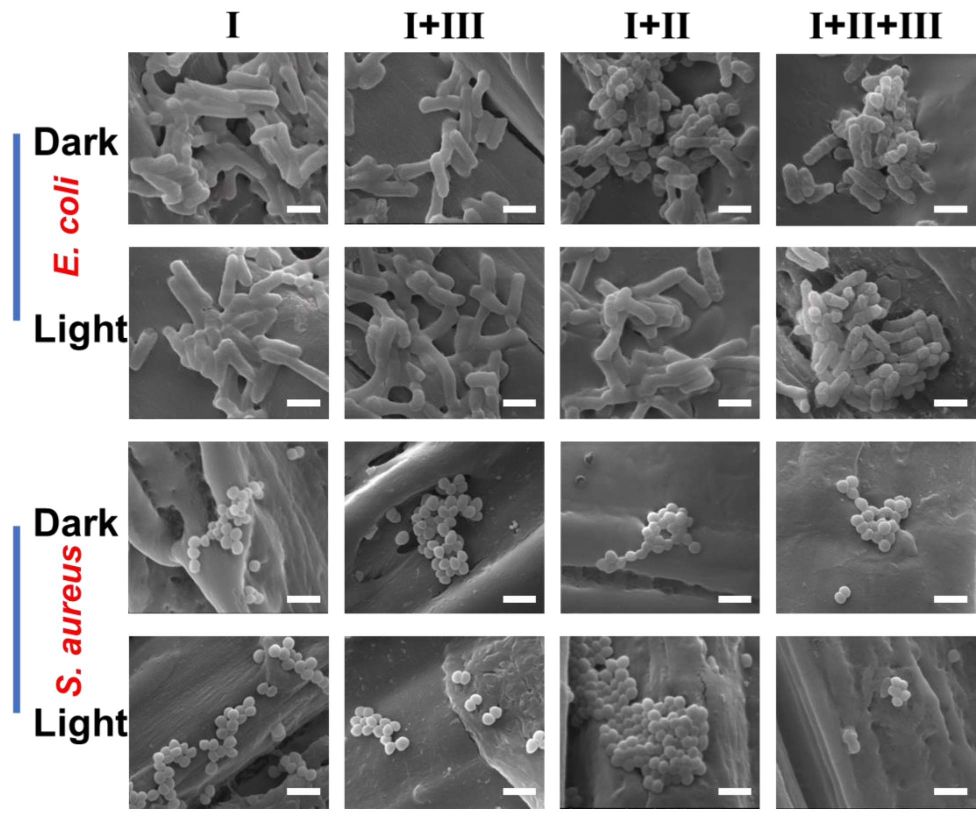

Fig. S24. SEM images of morphological changes of *E. coli* and *S. aureus* after different treatments. Scale bar=1 μm.

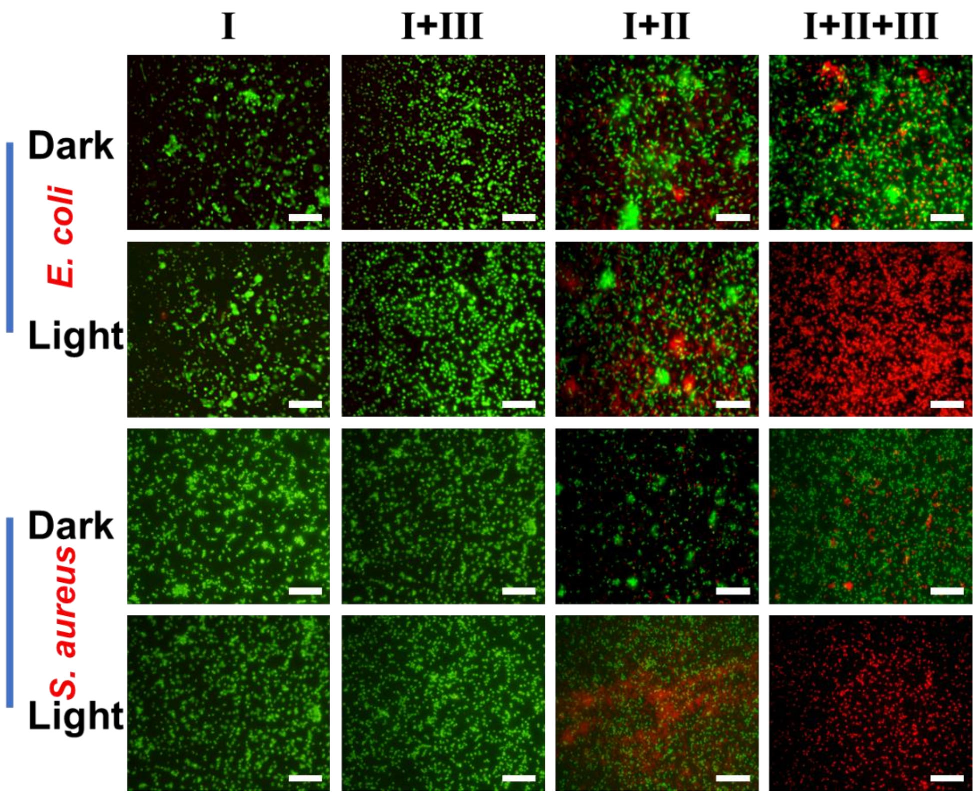

Fig. S25. Confocal fluorescence images of *E. coli* and *S. aureus* after different treatments. Scale bar=20 μm.

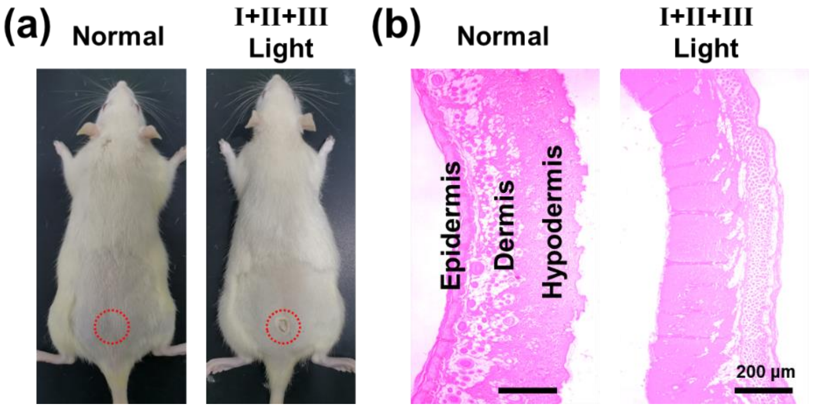

Fig. S26. (a) Photographs and (b) H&E staining of skin tissue after I+II+III Light treatment (660 nm laser, 0.5 W/cm^2, 5 min).

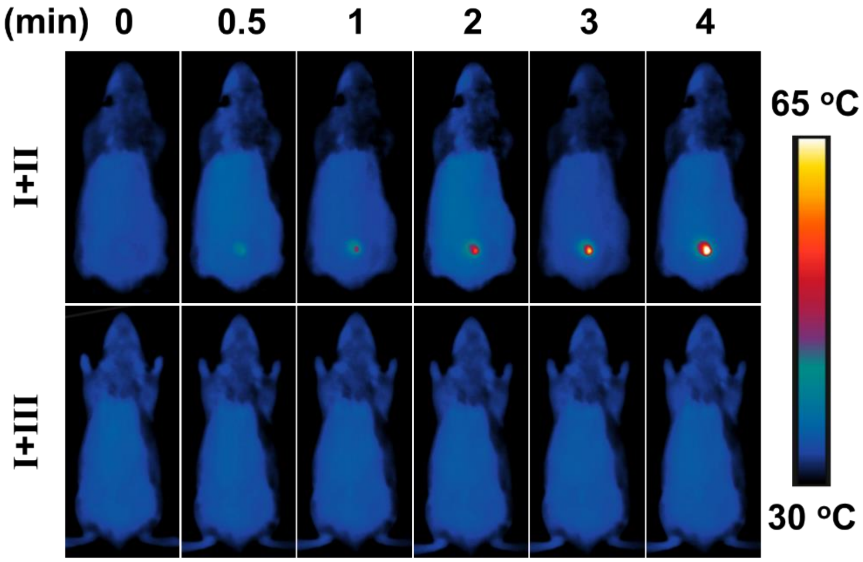

Fig. S27. Infrared thermal images at the wound sites of rats with different treatments under 660 nm laser irradiation (0.5 W/cm^2).

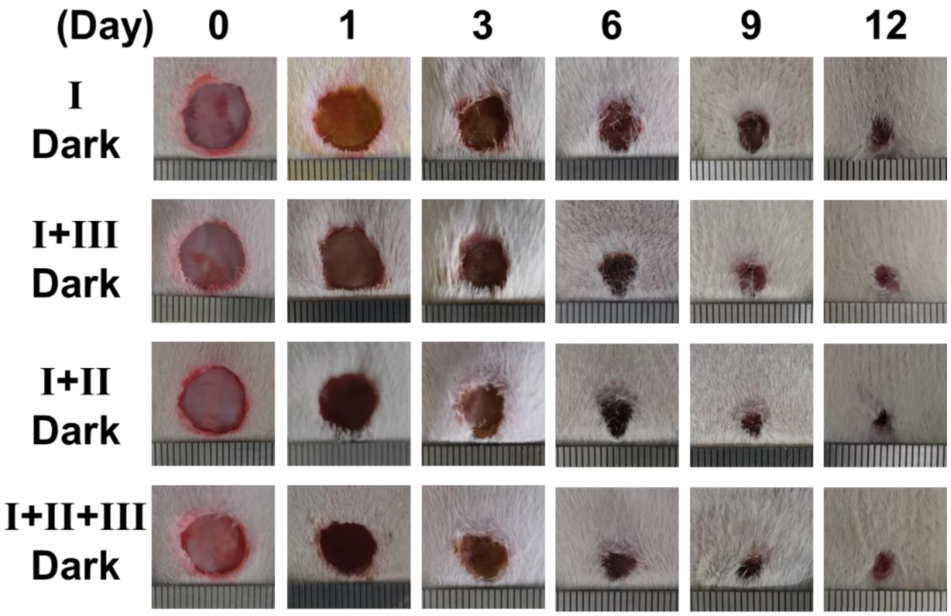

Fig. S28. Photographs of *S. aureus*-infected wounds treated under dark condition from day 0 to day 12.

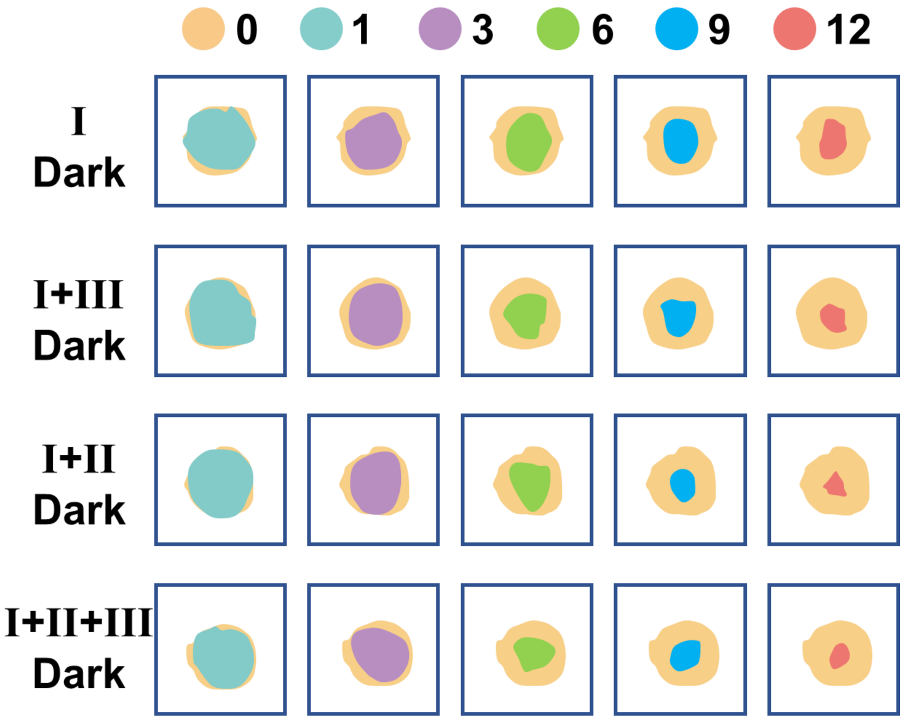

Fig. S29. Schematic images of corresponding wound contraction under dark conditions.

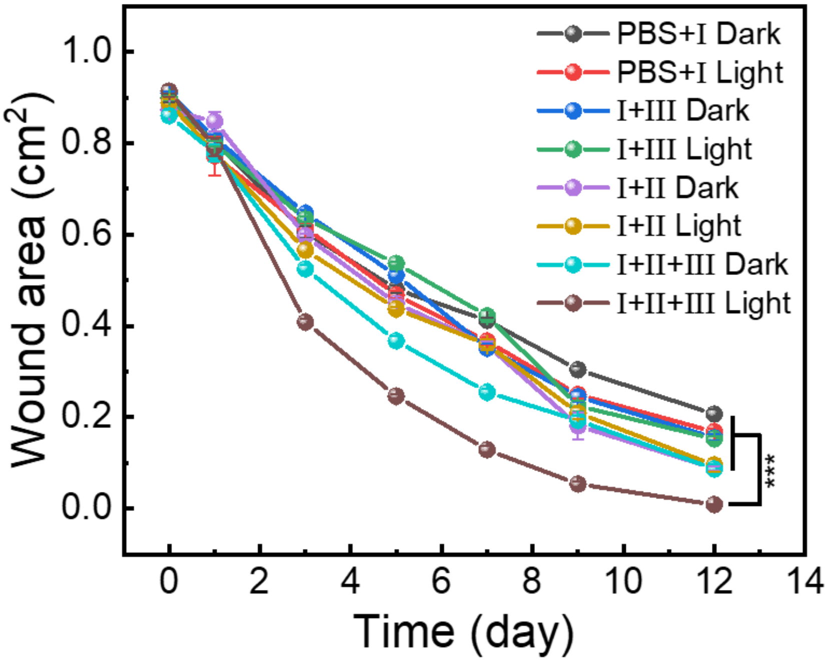

Fig. S30. Wound contraction curve of each group (n=3, ***$P<0.001$).

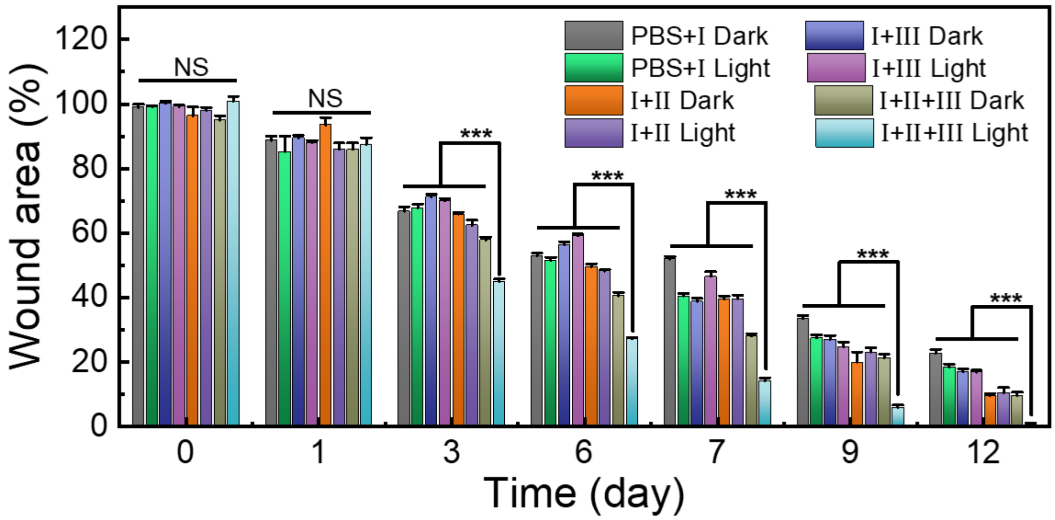

Fig. S31. Wound area for each group (n=3, ***P<0.001, and NS means not significant).

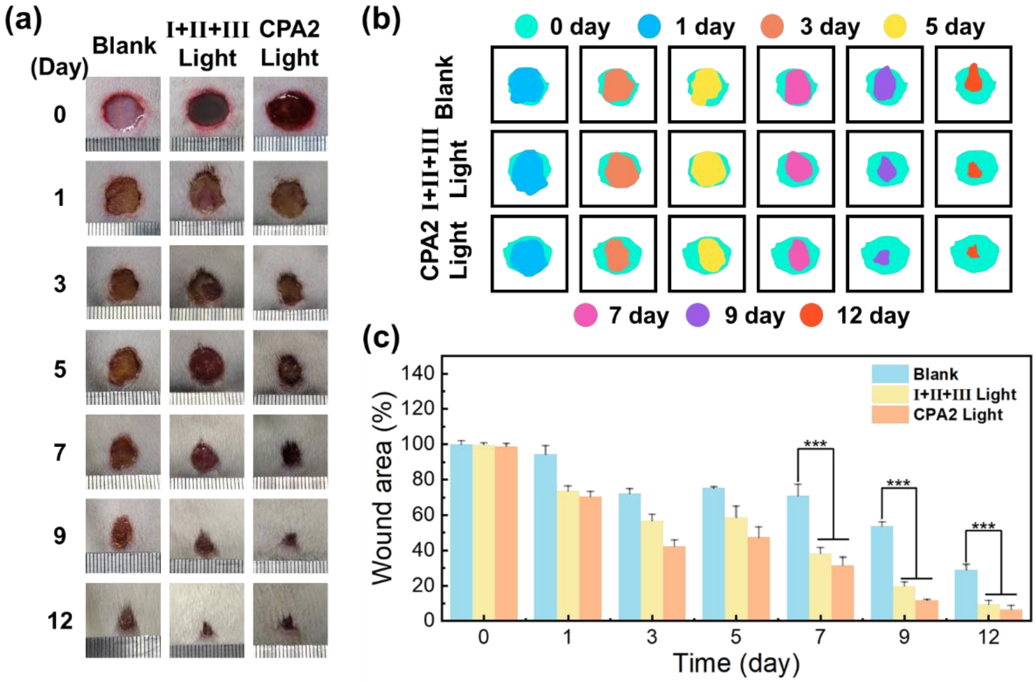

Fig. S32. (a) Photographs of *S. aureus*-infected wounds treated with Blank, I+II+III Light (660 nm laser, 0.5 W/cm^2, 5 min) and CPA2 Light (808 nm laser, 1.0 W/cm^2, 5 min) treatments from day 0 to day 12. (b) Corresponding schematic images and (c) area changes of wound contraction, n=3, ***P<0.001.

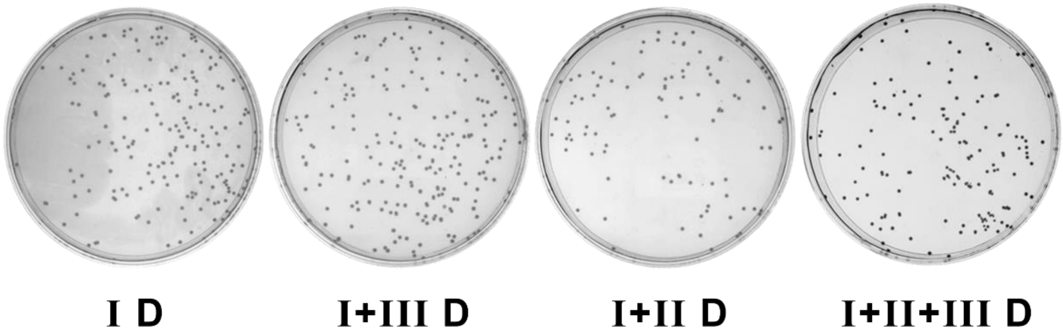

Fig. S33. Photographs of bacterial colonies on agar plates from wound sites on the first day with different treatments under dark condition (n=3).

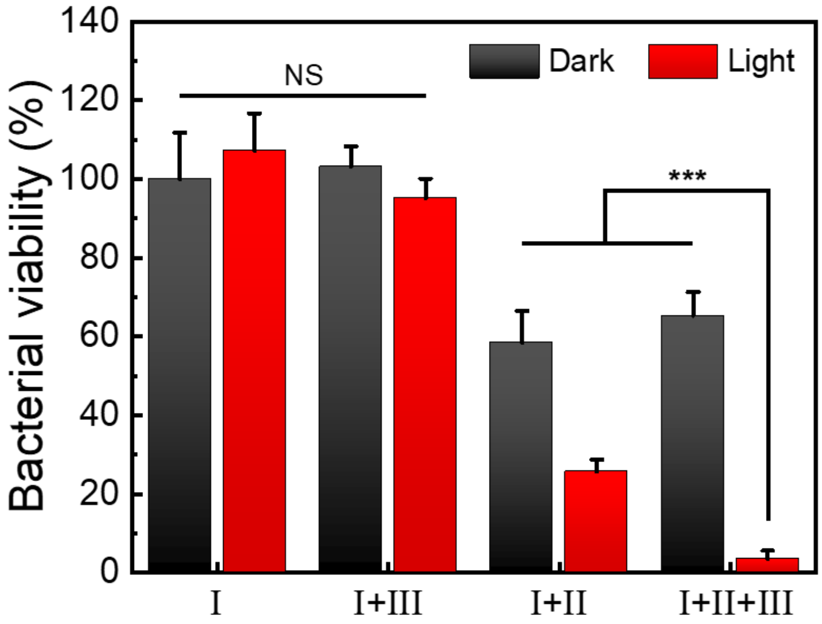

Fig. S34. Corresponding quantitative analysis of bacterial colonies on agar plates from wound sites on the first day after different treatments (n=3, ***P<0.001).

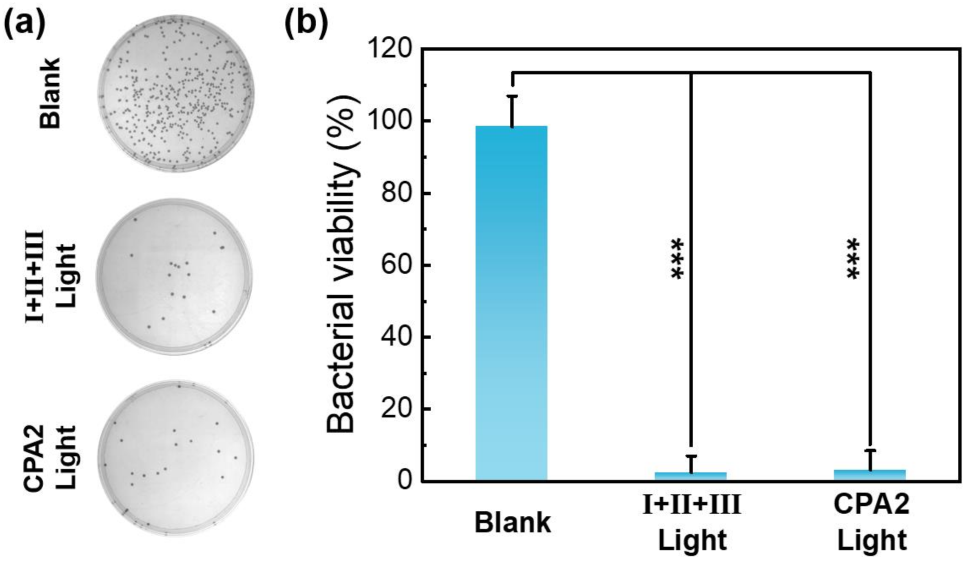

Fig. S35. (a) Photographs and (b) corresponding quantitative analysis of bacterial colonies on agar plates from wound sites on the 1st day with Blank, I+II+III Light (660 nm laser, 0.5 W/cm^2, 5 min) and CPA2 Light (808 nm laser, 1.0 W/cm^2, 5 min) treatments (n=3, ***P<0.001).

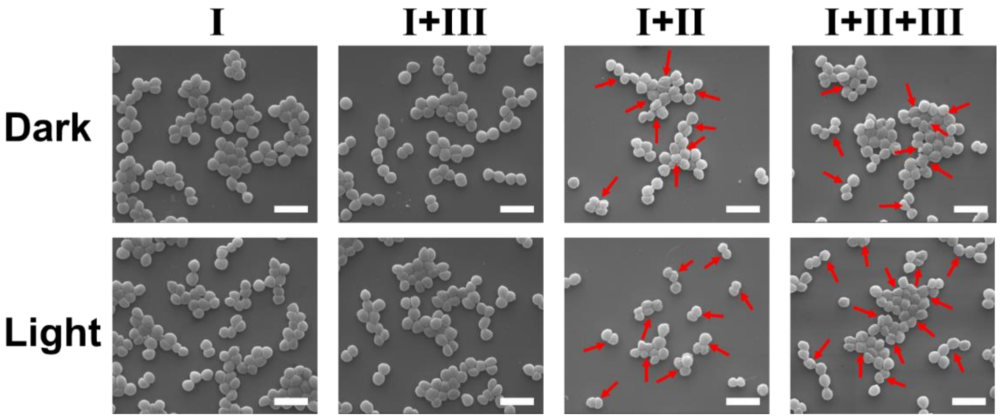

Fig. S36. SEM photographs of *S. aureus* after different treatments. Scale bar=1 μm.

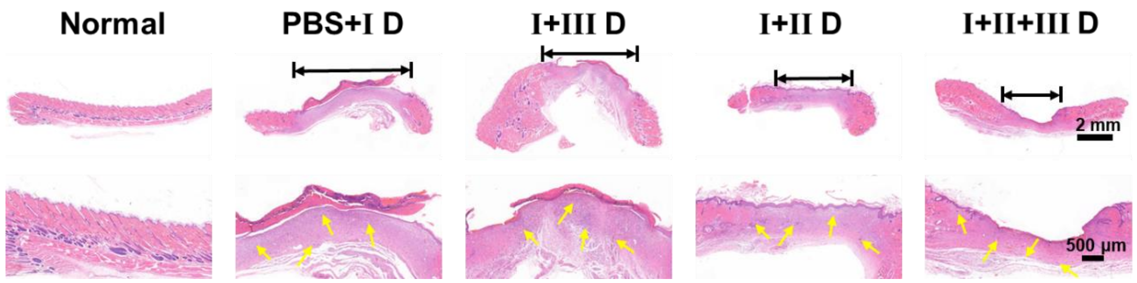

Fig. S37. H&E staining of healed skin tissues after different treatments under dark conditions.

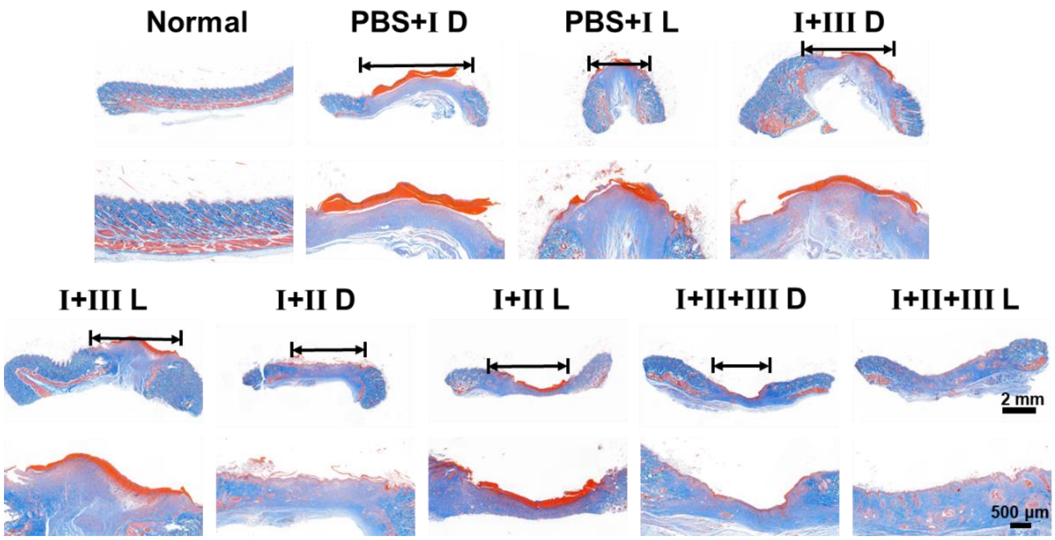

Fig. S38. Masson's trichrome staining of healed skin tissues after different treatments.

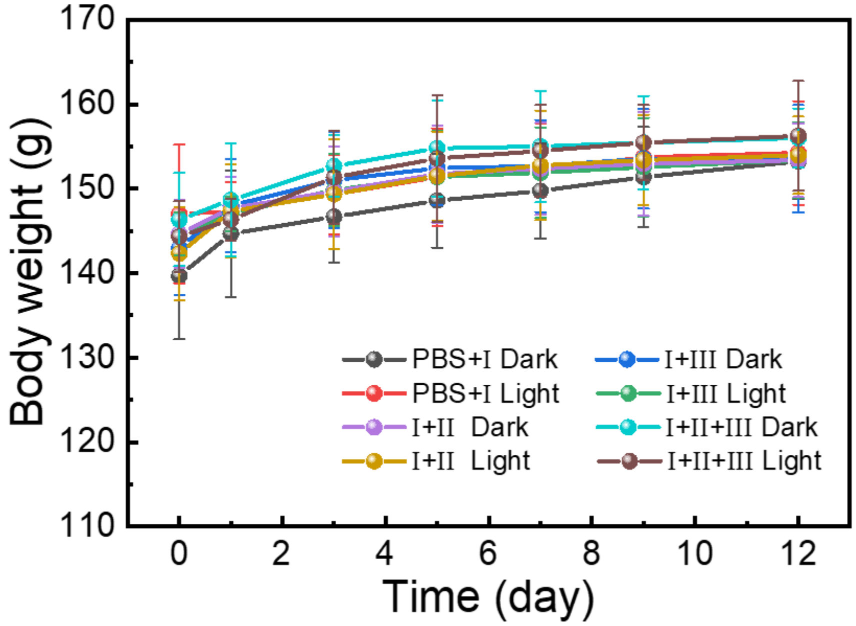

Fig. S39. Body weight changes of rats during different treatments (n=3).

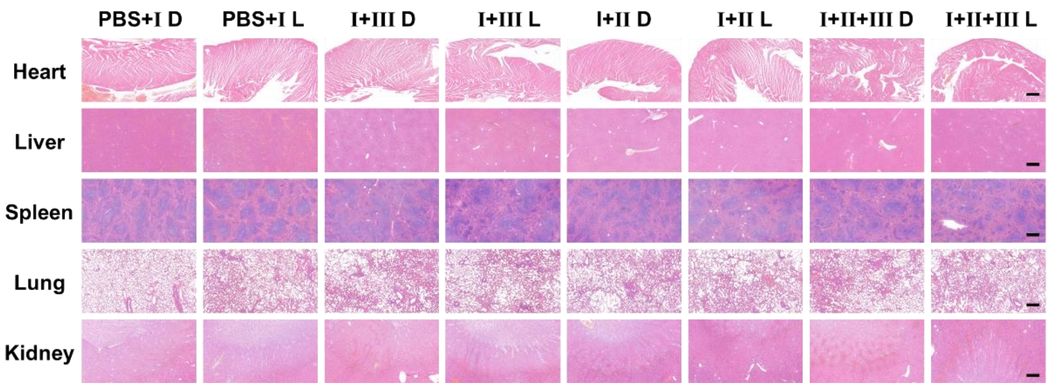

Fig. S40. H&E staining of main organs (heart, liver, spleen, lung, and kidney) was collected from rats in the different groups. Scale bar=500 μm.

SI References

1. B. Tang, W. L. Li, Y. Chang, B. Yuan, Y. Wu, M. T. Zhang, J. F. Xu, J. Li, X. Zhang, A supramolecular radical dimer: High-efficiency NIR-II photothermal conversion and therapy. *Angew. Chem. Int. Ed.* 58, 15526-15531 (2019).
2. Y. Sun, C. Chen, X. Wang, F. Zhang, S. Lu, X. Li, X. Suo, Z. Lin, Self-assembly of metallacages into centimeter films with tunable size and emissions. *J. Am. Chem. Soc.* 142, 17933-17937 (2020).
3. H. Hu, H. Wang, Y. Yang, J. F. Xu, X. Zhang, A bacteria-responsive porphyrin for adaptable photodynamic/photothermal therapy. *Angew. Chem. Int. Ed.* 134, e202200799 (2022).
4. X. Qi, Y. Huang, S. You, Y. Xiang, E. Cai, R. Mao, W. Pan, X. Tong, W. Dong, F. Ye, J. Shen, Engineering robust Ag-decorated polydopamine nano-photothermal platforms to combat bacterial infection and prompt wound healing. *Adv. Sci.* 9, 2106015 (2022).
5. B. Kim, H. Shin, T. Park, H. Lim, E. Kim, NIR-sensitive poly(3,4-ethylenedioxyselenophene) derivatives for transparent photo-thermo-electric converters. *Adv. Mater*. 25, 5483-5489 (2013).
6. W.-Z. Li, H. Chen, M.-N. Shen, Z. Yang, Z. Fan, J. Xiao, J. Chen, H. Zhang, Z. Wang, X.-Q. Wang, Chaotropic effect stabilized radical-containing supramolecular organic frameworks for photothermal therapy. *Small* 18, 2108055 (2022).
